# Argininosuccinate synthase 1 (ASS1) orchestrates arginine metabolism and ornithine production to modulate CHIKV infection

**DOI:** 10.1128/jvi.02098-24

**Published:** 2026-04-27

**Authors:** Nimisha Mishra, Mothe Sravya, Sonali Hanjankar, Anjali Singh, Yash Chaudhary, Ranjan Kumar Nanda, Sujatha Sunil

**Affiliations:** 1 Vector Borne Diseases Group, International Centre for Genetic Engineering and Biotechnology 28845, New Delhi, India; 2 TERI School of Advanced Studies (TERI-SAS) 121666 , New Delhi, India; 3 Translational Health Group, International Centre for Genetic Engineering and Biotechnology 28845, New Delhi, India; University of North Carolina at Chapel Hill, Chapel Hill, North Carolina, USA

**Keywords:** arginine, ASS1, CHIKV, arginase1, nitrite, ornithine, polyamines, STAT3, antiviral

## Abstract

Viruses reprogram the host metabolic machinery to ensure a continuous supply of macromolecules and energy for their own survival. Important cellular pathways are impacted during infection, resulting in changes in key metabolic precursors that influence infection outcomes. The present study was undertaken to evaluate the impact of L-arginine and argininosuccinate synthase 1 (ASS1), an important upstream enzyme of the arginine metabolism pathway, during chikungunya virus (CHIKV) infection in the human liver-derived Huh-7 cells. Using dose-dependent and time-course L-arginine supplementation experiments, we demonstrated that CHIKV exploits cellular arginine for enhanced viral replication. Loss-of-function and gain-of-function studies of ASS1, combined with nitric oxide donor treatments, revealed that arginine metabolism influences multiple downstream pathways, including ornithine synthesis, proline metabolism, and nitric oxide production during CHIKV infection. We further examined the relationship between ASS1 expression and STAT3 signaling in the context of viral infection. Our results demonstrate that exogenous L-arginine supplementation and ASS1 overexpression enhance CHIKV replication in Huh-7 cells. Conversely, ASS1 silencing resulted in >95% reduction in viral titers. Mechanistically, ASS1 modulated arginase 1 activity, affecting ornithine production and downstream metabolites while also influencing the cellular nitroso-redox environment. Additionally, ASS1 expression affected STAT3 levels and its subcellular localization: ASS1 overexpression correlated with reduced nuclear STAT3 accumulation and increased viral replication, whereas ASS1 depletion promoted STAT3 nuclear translocation and restricted viral infection. These findings reveal a complex interplay between arginine metabolism, innate immune signaling, and CHIKV replication, identifying ASS1 as a potential regulatory node in CHIKV-host interactions.

**IMPORTANCE:**

Metabolic reprogramming of the host is crucial for the virus to establish itself within the cell, and in this process, the virus hijacks several host metabolic pathways. We examined the role of an important arginine metabolizing enzyme, human argininosuccinate synthase (ASS1), during CHIKV infection in liver cells through silencing and overexpressing ASS1 and by L-arginine supplementation. We demonstrate that ASS1 favors CHIKV replication and also plays important roles in several downstream cellular processes during virus infection. This study further deepens our understanding of the significance of the crucial metabolites involved in the arginine metabolism pathway during CHIKV infection and how CHIKV exploits the specific pathway to enhance its replication.

## INTRODUCTION

The arginine metabolism pathway in mammals, primarily responsible for restoring the plasma arginine level and production of NO, is a vital amino acid metabolism pathway (1, 2). Arginine is involved in multiple metabolic processes, such as the elimination of nitrogenous products via the urea cycle and the Citrulline-NO cycle (3, 4). Arginine can be used as a dietary supplement, and depletion of arginine is a novel antimetabolite strategy for treating arginine-dependent cancers (5, 6). Arginine can also act as a signaling molecule and plays a crucial role in cancer metabolism, as an epigenetic regulator, and as an immunomodulator (7).

Mammalian arginine metabolism is highly complex, involving a multitude of enzymes that either compete or interact with one another to utilize arginine as a substrate (8). Multiple isozymes are involved in arginine metabolism, and their expression can be altered in response to infection and diseases. Two such cytosolic enzymes, that is, argininosuccinate synthase 1 (ASS1) and argininosuccinate lyase (ASL), are responsible for the biosynthesis of arginine from citrulline, where aspartate acts as a co-substrate (9). These enzymes provide the essential environment in the cell for the availability of the final product of these reactions, namely, L-arginine, which stands at a crossroads for multiple pathways and is a common substrate for arginase 1 and nitric oxide (NO) (10). It is noteworthy that L-arginine, although endogenously produced through the above metabolism, is not entirely sufficient for the generation of NO under conditions of high demand, such as stressful situations (8). Furthermore, it is also known that providing exogenous arginine does not directly translate to enhanced NO production, underscoring a delicate balance described as the “arginine paradox” (11).

ASS1 exists as a homotetrameric enzyme that catalyzes the production of argininosuccinate from aspartate and citrulline and has been extensively studied for its vital role in hepatic urea production, polyamine synthesis, and creatinine synthesis (12). It is also a target for multiple viral pathogens such as Herpes Simplex virus (HSV), human cytomegalovirus (HCMV), and Kaposi’s sarcoma-associated herpesvirus (KSHV), playing multiple roles that can either create a conducive metabolic state for promoting viral replication or aid in activating the innate immune signaling toward oncogenic transformation of the host cell (13, 14).

Alphaviruses, such as chikungunya virus (CHIKV), are enveloped positive single-stranded RNA viruses that infect a plethora of host cells and cause pathogenesis ranging from acute to chronicity of the disease (15, 16). The viruses encode proteins that are involved in viral assembly as well as their replication. The structural proteins include capsid, E3, E2, E1, and 6K, while the nonstructural proteins (nsPs1-4) exhibit different biochemical functions. The replication machinery of the virus consists of nsP1, which shows methyl and guanyl transferase activity, nsP2 has metabolic enzymes such as helicase and protease, nsP3 macrodomain has ADP ribosyl hydrolase activity, and nsP4 is an RNA-dependent RNA polymerase, among others that are essential for viral replication (17–21). Several studies have provided insights into the viral hijacking of the host cellular machinery for its survival and spread. One important aspect to be considered with respect to host cell modulation during virus infection is the functional relevance of the infected cell type, which is known to be virus-specific. Cellular tropism of CHIKV has identified a subset of cells associated with persistent infection, such as muscle cells, fibroblasts, epithelial cells, lymphoid cells, and macrophages (17, 22–25). Among the different cellular and organ tropisms, the liver is defined as one of the primary target organs of CHIKV, which supports virus replication along with joints, muscles, brain, and spleen (22–26).

In the present study, we analyzed the role of human ASS1 during CHIKV infection using loss-of-function assays, gain-of-function assays, and L-arginine supplementation in the Huh-7 liver cell line. We demonstrated that liver cells exhibit the “arginine paradox” during CHIKV infection based on the exogenous supplementation of L-arginine to the cells upon infection. We further evaluated the mode of action of ASS1 toward metabolizing arginine in several pathways it is known to divert into, such as polyamine, proline, and NO production. We finally evaluated the role of ASS1 in regulating STAT3 expression during CHIKV infection in liver cells. Our results illustrate for the first time the possible mechanism of how CHIKV hijacks the host arginine metabolism pathway to enhance its replication in liver cells.

## RESULTS

### Arginine supplementation in liver cells promotes CHIKV infection

**Fig 1:**
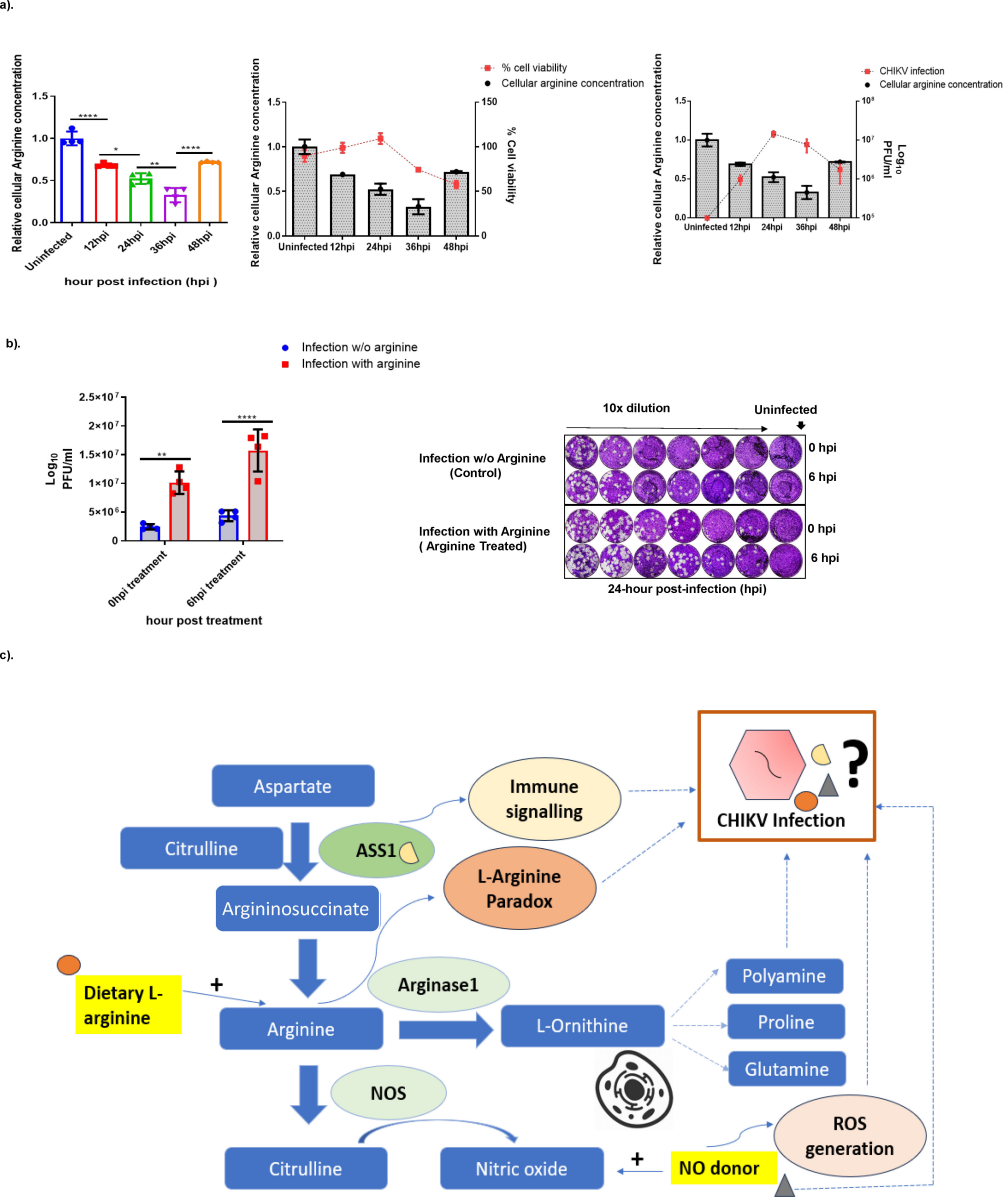
Arginine metabolism in human liver cells during CHIKV infection. (**a**) Cellular arginine concentration (in nmol/μL) during CHIKV infection at different time points. Huh7 cells were seeded in 12-well plates. After reaching 80% confluency, the cells were infected with CHIKV at a multiplicity of infection (MoI) of 0.1. Cells were lysed in RIPA buffer, and the supernatant was then used to measure cellular arginine concentration. Absorbance was measured at 450 nm, and cellular arginine concentration was expressed in nanomoles per microliter, showing relative changes on the left y-axis. The x-axis represents the time point after CHIKV infection. The graphs were plotted after normalizing arginine levels vis-à-vis uninfected cells at the same time points of infection. Data are presented as mean ± SD and analyzed with one-way ANOVA (*P* = 0.0001), followed by Tukey’s multiple comparison test. Significant differences are marked by stars; ns for non-significant, * for *P* < 0.05, ** for *P* < 0.01, *** for *P* < 0.001, and **** for *P* < 0.0001. The experiment was performed three times. Middle panel: MTT assay evaluating the cytotoxicity of CHIKV infection over time, that is, uninfected, 12 hpi, 24 hpi, 36 hpi, and 48 hpi. One-way ANOVA and Tukey’s multiple comparison test were used for analysis. The x-axis displays the time point after infection, while the left y-axis indicates the relative cellular arginine concentration (in nmol/μL) during CHIKV infection, and the right y-axis shows the percentage of cell viability. Third panel: Quantification of CHIKV titer using plaque assay: Huh7 cells were seeded in 12-well plates. After reaching 80% confluency, cells were infected with CHIKV at an MoI of 0.1. Culture filtrate was collected at specified time points (12, 24, 36, and 48 hpi), and a plaque assay was performed in Vero cells. The experiment was repeated several times. The one-way ANOVA and Tukey’s multiple comparison test were used for analysis. The x-axis displays the time point after infection. The left y-axis indicates the relative cellular arginine concentration (in nmol/μL) during CHIKV infection, and the right y-axis shows the CHIKV titer (PFU/mL). (**b**) Kinetics of virus infection upon arginine supplementation assessed via plaque assay. Huh7 cells were seeded in 12-well plates and infected with CHIKV at 0.1 MOI upon reaching 80% confluency. After infection, serum-free DMEM medium was replaced with arginine-enriched media at 0 and 6 hpi. The culture filtrate was collected at specified time points and conditions, and infectious virions were quantified using a plaque assay in Vero cells. The graph shows the virus titer 24 h post-infection after arginine supplementation, with the “Time of addition of arginine” indicated at 0 and 6 hpi. Error bars represent standard deviation. Statistical analysis was performed using two-way ANOVA and Tukey’s multiple comparison tests. Stars denote significant differences between the infected control (arginine-) and the arginine-supplemented (arginine+) infected samples; ns for non-significant, ** for *P* = 0.001, and **** for *P* < 0.0001. The right panel shows representative plaque plates for both arginine-treated infected samples (0 and 6 hpi) and untreated infected controls, prepared using 10-fold dilutions. (**c**) Schematic illustration of the hypothetical model used to investigate arginine metabolism and related pathways in humans during CHIKV infection. Arginine directly serves as a precursor for ornithine and NO. It is also derived from sources like proline and glutamine. L-arginine is metabolized through two primary pathways: the nitric oxide synthase (iNOS) pathway, which produces citrulline and NO, and the arginase pathway, which generates ornithine and urea. This model emphasizes the roles of specific metabolites and enzymes involved in these pathways during CHIKV infection. Silencing the ASS1 gene is used to evaluate its impact on CHIKV replication, alongside factors such as dietary arginine supplementation, the addition of NO donors, and ROS production.

L-Arginine is a semi-essential amino acid in humans with remarkable regulatory and metabolic versatility. It serves as a major metabolic nexus for the generation of a diverse range of metabolites such as nitric oxide (NO), polyamines, creatinine, agmatine, glutamate, proline, and urea. In most mammalian cells, arginine is generated through the arginine-citrulline cycle to meet the cellular requirement for downstream metabolites; however, during stress conditions, including viral infections, host arginine metabolism is dysregulated (14). To evaluate the role of arginine metabolism during CHIKV infection, we monitored intracellular arginine levels in Huh-7 cells over the course of infection. Measurements of arginine levels in CHIKV-infected cells revealed significant changes over time (Fig. 1a, first panel). Cell viability analysis revealed minimal cell death up to 24 hpi, with mortality increasing to 25% at 36 hpi and approximately 50% at 48 hpi (Fig. 1a, middle panel).

We observed depletion of arginine levels coinciding with active viral replication at 12, 24, and 36 hpi (Fig. 1a, third panel), suggesting substantial arginine consumption during viral replication. Notably, at 48 hpi, cellular arginine levels increased significantly despite ongoing cell death and reduced viral titers. We reasoned that this increased arginine concentration at 48 hpi, concurrent with 50% cell death, likely results from several interconnected mechanisms that disrupt normal arginine homeostasis. For instance, during infection-induced cell death, key arginine-metabolizing enzymes such as argininosuccinate synthase 1 (ASS1), arginase 1 (ARG1), and nitric oxide synthase are inhibited, creating a metabolic bottleneck that reduces arginine consumption. Simultaneously, cell death processes cause membrane dysfunction and loss of subcellular compartmentalization, which disrupts amino acid transport gradients while cellular proteolysis liberates arginine from protein structures (27–29). The convergence of reduced metabolic consumption, impaired cellular export, and enhanced proteolytic release creates conditions that favor intracellular arginine accumulation, even as overall cell viability declines (Fig. 1).

**Fig 2:**
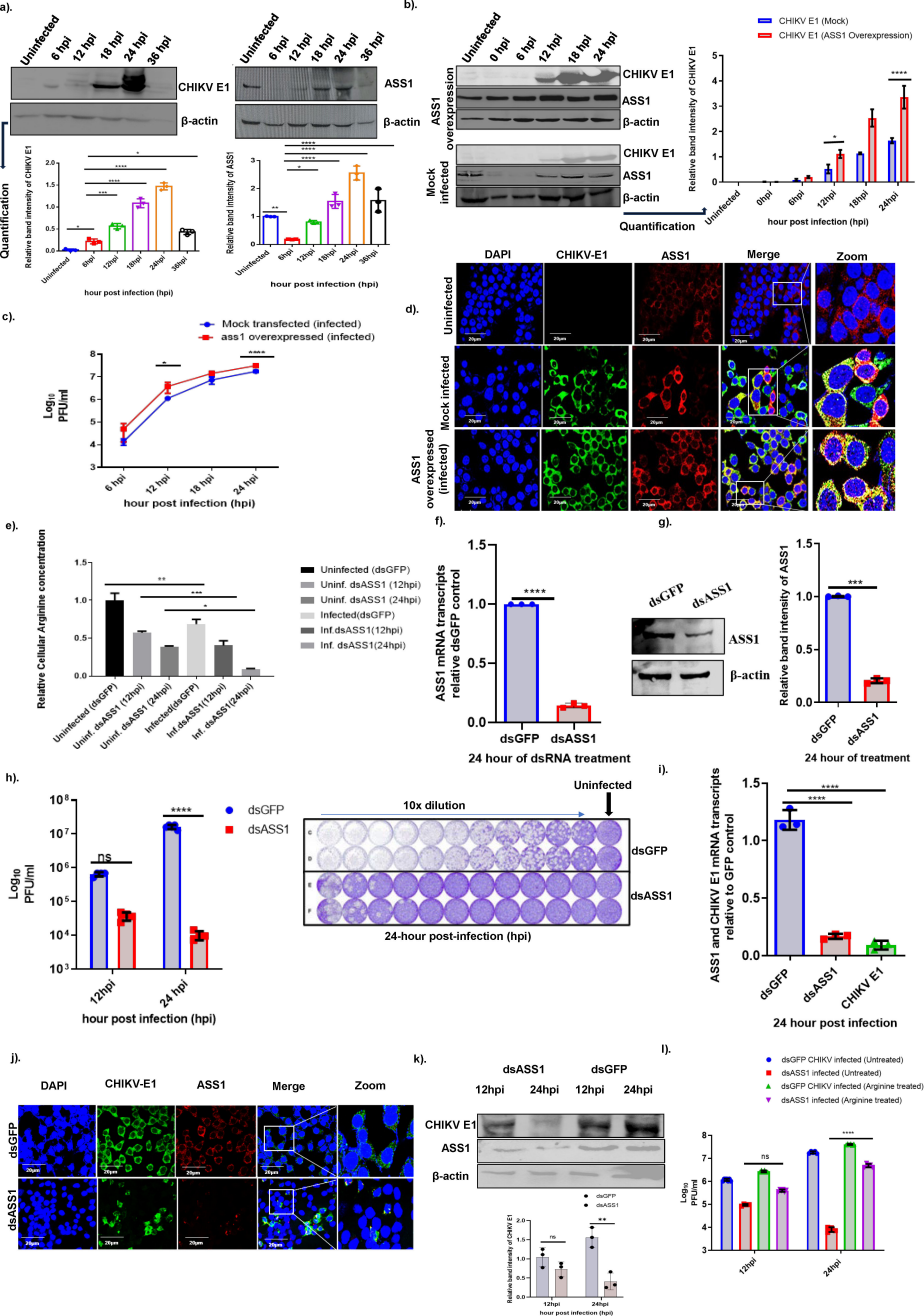
Regulation of ASS1 during Chikungunya virus infection. (**a**) Western blotting depicting the expression of CHIKV E1 and ASS1 protein and its quantification over time during infection. At specified time points, CHIKV-infected Huh7 cells were lysed in RIPA buffer, and the supernatants were analyzed by immunoblotting with β-actin as a loading control. A representative blot from three biological replicates is shown. The bar plot at the bottom measures the band intensities of CHIKV E1 and ASS1 after normalization to β-actin during infection. Statistical significance was assessed using one-way ANOVA and Tukey’s multiple comparison test, with stars indicating significant differences; * for *P* = 0.01, and **** for *P* < 0.0001 as depicted in the figure. (**b**) Western blot results showing the expression of the ASS1 and CHIKV E1 proteins under the ASS1 overexpression condition, with mock transfection (using an empty pCMV vector) as a control. Huh7 cells were grown to 80% confluency and then transfected with either the pCMV-ASS1 plasmid or an empty pCMV vector as a mock control. After 24 h, the cells were infected with CHIKV at an MOI of 0.1. Samples were collected at specific time points: 0, 6, 12, 18, and 24 h post-infection (hpi). At each point, culture filtrates were used for plaque assays, and cell lysates were subjected to immunoblotting, with β-actin serving as a loading control. It is worth noting that "0 hpi” in our experimental setup indicates 2 h after infection, not an uninfected state. This time point marks the period when the virus enters host cells and is biologically different from the uninfected control. The accompanying bar graph illustrates the relative band intensities of CHIKV E1 at various time points under mock and ASS1 overexpression conditions. Data from three independent experiments are displayed, presented as mean ± SD. Statistical significance was assessed using two-way ANOVA and Tukey’s multiple comparison test, with stars indicating significant differences; * for *P* = 0.01, and **** for *P* < 0.0001. (**c**) Assessment of CHIKV virus titers in Huh7 cells was conducted using a plaque assay during ASS1 overexpression at 6, 12, 18, and 24 hpi, compared to mock-transfected, CHIKV-infected cells. Data analysis involved a two-way ANOVA, followed by Sidak’s multiple-comparison test. Significant differences between the mock-infected control and the ASS1-overexpressed infected samples are marked with stars; * for *P* = 0.01 and **** for *P* < 0.0001. The experiment was performed with three biological replicates. (**d**) Confocal fluorescence microscopy was employed to analyze ASS1 and CHIKV E1 expression, stained with Alexa Fluor 594 (red), and CHIKV E1, labeled with Alexa Fluor 488 (green), in uninfected cells, CHIKV-infected cells, and ASS1-overexpressing cells. The nucleus was visualized with DAPI (blue). The experiment involved uninfected Huh7 cells, CHIKV-infected cells for 24 h, and cells transfected with the pCMV-ASS1 plasmid and then infected with CHIKV. The cells were fixed and further stained with DAPI, anti-ASS1, and anti-CHIKV E1 antibodies. The merged images display both red and green channels, with scale bars indicating 20 µm. Representative images are shown. (**e**) Relative cellular arginine levels during ASS1 knockdown, that is, dsASS1 treatment, and dsASS1+ infection compared to uninfected dsGFP control. The experiment included uninfected dsGFP-treated cells, uninfected dsASS1-treated cells, infected dsGFP cells, and infected dsASS1 cells to measure cellular arginine levels (as described in the Materials and Methods section). The experiment was conducted three times. Absorbance was measured at 450 nm, and cellular arginine levels were expressed in nanomoles per microliter. Relative changes in the arginine level are shown on the left y-axis, and experimental conditions are plotted on the x-axis. One-way ANOVA and Tukey’s multiple comparison test were used for the analysis. Data are presented as mean ± SD. (**f and g**) Reverse transcription (RT)-qPCR and western blot demonstrate the knockdown effect at both the mRNA and protein levels. dsGFP serves as the negative control in uninfected conditions. Huh7 cells were cultured to about 80% confluence and then treated with ASS1 dsRNA or dsGFP control. The cells were harvested using TRIzol for RNA extraction, while the RIPA buffer was used to lyse the cells for immunoblotting. (**h**) ASS1 knockdown decreases viral yield. The knockdown was carried out as described in (**f and g**); the cells were treated with either dsGFP or dsASS1, followed by infection with CHIKV at an MOI of 0.1. At 12 and 24 h post-infection, the viral yield was measured by plaque assay. Significant differences are marked by stars, **** for *P* < 0.0001 and ns for non-significant and were obtained through two-way ANOVA with Tukey’s multiple comparison test. Significance was determined by comparing the treated groups (dsASS1) to their respective controls (dsGFP). The right panel shows the plate layout of the plaque assay performed on Vero cells with 10-fold serial dilutions. The culture filtrate was serially diluted, incubated with Vero cells, and plaque assays were performed. (**i**) Reverse transcription (RT-qPCR) demonstrating the knockdown of ASS1 and CHIKV E1 at the mRNA level during infection. Huh7 cells were transfected with dsGFP or dsASS1, and 24 h after transfection, CHIKV infection was performed. The cells were lysed in TRIzol reagent to measure ASS1 and CHIKV E1 mRNA levels at 24 hpi using quantitative qRT-PCR. The experiments were conducted independently three times. Statistical significance was assessed using one-way ANOVA, followed by Tukey’s multiple comparison test; where *P*-value **** ≤0.0001. (**j**) Immunofluorescence imaging of ASS1, stained with Alexa Fluor 594 (red), and CHIKV E1, probed with Alexa Fluor 488 (green), demonstrates protein expression in the control dsGFP condition and during dsASS1 knockdown in CHIKV-infected Huh7 cells. The nucleus was stained with DAPI (blue). (**k**) The top panel shows the western blot depicting the CHIKV E1 protein expression in cells treated with dsASS1 compared to those treated with dsGFP under infected conditions. The first two lanes of the immunoblot show dsASS1 (infected) conditions at 12 and 24 hpi, while the last two lanes show dsGFP (infected) controls at 12 and 24 hpi. The bottom panel is the densitometric quantification of CHIKV E1 protein normalized to β-actin in dsGFP- and dsASS1-treated conditions. Statistical significance was determined using one-way ANOVA, where *P*-value ** = 0.0011. (**l**) CHIKV titers were measured by plaque assay at 12 and 24 hpi, with and without arginine supplementation. The experiment involved infecting Huh7 cells with CHIKV; one group was treated with 0.1 mM arginine, while another was not. Additionally, Huh7 cells treated with dsASS1 were infected similarly, with one group receiving arginine and the other not. After 12 and 24 h, the culture media were collected to evaluate viral yield using plaque assays. *P*-value: **** ≤0.0001 was determined by two-way ANOVA, followed by Tukey’s multiple comparison test. Each experiment was independently repeated at least three times.

Furthermore, to assess the functional significance of arginine availability during CHIKV infection, we supplemented cell cultures with exogenous L-arginine and evaluated viral titers at 24 hpi. The complete DMEM medium contains L-arginine at 0.084 g/L, which served as the baseline condition for both uninfected and infected Huh7 cells throughout our experiments. Supplementation with an additional 0.1 mM L-arginine, administered at 0 and 6 hpi, was carried out to coincide with early viral replication events. When arginine supplementation was provided immediately upon infection (0 hpi), we observed a 4.4-fold increase in viral progeny when compared with unsupplemented cultures. Conversely, when arginine was supplemented at 6 hpi, viral titers increased by 3.7-fold relative to controls (*P* < 0.0001) (Fig. 1b). Plaque assay plates with 10-fold serial dilutions of viral supernatants show increased plaque formation in arginine-treated samples versus controls, that is, without arginine (Fig. 2b, right panel). These results clearly demonstrate the critical role of arginine availability during CHIKV infection and indicate that the timing of supplementation influences the magnitude of enhancement, with earlier supplementation providing greater benefit for viral replication.

The above results prompted us to elucidate the mechanisms underlying arginine’s role in CHIKV pathogenesis in greater depth, considering this amino acid serves as a precursor to several metabolites that provide critical resources for both viruses and the host, such as NO, ornithine, and polyamines. Given that cellular arginine synthesis occurs primarily through argininosuccinate synthase 1 (ASS1), we hypothesized that this enzyme plays a pivotal role in modulating downstream pathways associated with arginine metabolism and consequently impacts CHIKV replication. To test this hypothesis, we employed a comprehensive experimental approach involving ASS1 silencing, exogenous arginine supplementation, and NO donor treatments to investigate arginine-associated pathways, including polyamine biosynthesis, NO synthesis, reactive oxygen species (ROS) activation, and STAT3 signaling during CHIKV infection in liver cells (Fig. 1c).

### The arginine metabolizing enzyme, ASS1, is regulated during CHIKV infection in liver cells

In the biosynthesis of arginine, argininosuccinate synthase 1 (ASS1) catalyzes the rate-limiting step in the citrulline-NO cycle. To examine the temporal dynamics of ASS1 expression during CHIKV infection, we evaluated its protein expression at multiple time points post-infection, specifically at 6, 12, 18, 24, and 36 hpi. As shown in Fig. 2a (first blot), CHIKV E1 became detectable by 6 hpi and rose steadily thereafter, with a pronounced accumulation at 18–24 hpi and a slight decline by 36 hpi. Densitometric quantification normalized to β-actin showed significant increases relative to uninfected controls from 12 hpi onward, peaking at 24 hpi (statistics indicated on the graph; *P* values as denoted by stars). These results verify progressive viral protein production across the first 24 h of infection.

ASS1 exhibited a time-dependent, infection-associated induction (Fig. 2a, second blot). In uninfected cells, basal ASS1 expression was detected. Following infection, ASS1 levels remained relatively low at 6 hpi but increased markedly at 12 hpi and 18 hpi and peaked at 24 hpi. Densitometric analysis demonstrated that ASS1 expression at 24 hpi was significantly elevated compared to 6 h of infection. A decrease was observed at 36 hpi, mirroring the trend seen with CHIKV E1 expression (Fig. 2a, right graph). β-actin levels remained relatively stable across all time points, confirming equal protein loading. It was, however, noted that at 36 hpi, the actin expression level also decreased, suggestive of cell death post 24 hpi.

Next, to test whether elevating ASS1 alters viral protein accumulation, we repeated the time course with ectopic ASS1 expression and a matched mock control (Fig. 2b; time points: uninfected, 0, 6, 12, 18, and 24 hpi). Multiple independent western blot experiments verified robust ASS1 overexpression relative to mock across all time points, which was accompanied by higher CHIKV E1 protein levels throughout infection, with the largest increase at 24 hpi (bar graph; ****P at 24 hpi). Consistently, plaque assays showed that ASS1 overexpression modestly increased infectious virus production at later time points, yielding an ~2-fold enhancement at 24 hpi (Fig. 2c). Confocal microscopy further revealed basal ASS1 expression in uninfected cells without detectable E1 (Fig. 2d, top row), marked cytoplasmic signals for both proteins with overlap (middle row) during infection, and markedly intensified, overlapping ASS1 and E1 signals in ASS1-overexpressing infected cells (bottom row; scale bars, 20 µm), corroborating the immunoblot results and indicating that ASS1 upregulation promotes CHIKV E1 accumulation at the cellular level.

To further establish the functional importance of ASS1 in CHIKV infection, we performed loss-of-function experiments using dsRNA-mediated knockdown of ASS1. First, we measured cellular arginine levels under different experimental conditions. Compared to uninfected dsGFP control cells, treatment with dsASS1 alone (without infection) significantly reduced cellular arginine levels. CHIKV infection in dsGFP-treated cells slightly decreased arginine levels, but the most substantial 11-fold reduction was observed in dsASS1-treated infected cells (Fig. 2e). These results were analyzed using one-way ANOVA with Tukey’s multiple comparison test and are presented as mean ± SD from three biological replicates. The data demonstrate that ASS1 contributes significantly to maintaining cellular arginine pools, which are further depleted during CHIKV infection. Reverse transcription quantitative PCR (RT-qPCR) and western blot analysis confirmed efficient knockdown of ASS1 at both the mRNA and protein levels. Compared to dsGFP-treated controls, dsASS1 treatment resulted in a dramatic reduction in *ASS1* mRNA expression (****P < 0.0001) (Fig. 2f). Western blot analysis corroborated these findings, showing substantially decreased ASS1 protein levels in dsASS1-treated cells compared to dsGFP controls (Fig. 2g).

Having established effective ASS1 knockdown, we next examined its impact on CHIKV replication. Huh7 cells were treated with dsASS1 or dsGFP control, followed by CHIKV infection at 0.1 MOI. Viral titers in culture supernatants were measured by plaque assay at 12 and 24 hpi. ASS1 knockdown significantly reduced viral yield at 24-h time point compared to dsGFP controls (Fig. 2h, left graph; ****P < 0.0001 at 24 hpi). Representative plaque assay plates showing 10-fold serial dilutions of viral supernatants clearly demonstrate reduced plaque formation in dsASS1-treated samples compared to dsGFP controls (Fig. 2h, right panel). RT-qPCR analysis further confirmed that *ASS1* knockdown reduced *CHIKV E1* mRNA levels at 24 hpi. Compared to dsGFP-treated infected cells, dsASS1-treated infected cells showed significantly decreased *CHIKV E1* mRNA expression, while *ASS1* mRNA levels were also substantially reduced (****P < 0.0001) (Fig. 2i). These results demonstrate that ASS1 knockdown impairs viral replication at the transcriptional level. Immunofluorescence microscopy provided visual confirmation of these findings. In dsGFP-treated infected cells, both ASS1 (red) and CHIKV E1 (green) signals were readily detectable in the cytoplasm (Fig. 2j, top row). In contrast, dsASS1-treated infected cells showed markedly reduced ASS1 signal and a corresponding decrease in CHIKV E1 expression (Fig. 2j, bottom row). Western blot and the densitometry analysis of infected cells treated with dsGFP or dsASS1 at 24 hpi confirmed these results. CHIKV E1 protein levels were significantly lower in dsASS1-treated cells compared to dsGFP controls at 24 h, and ASS1 protein was effectively depleted by dsRNA treatment (Fig. 2k, upper blots). It should be noted that the western blot analysis was performed using cell lysate and depicts protein expression of E1 as opposed to the plaque data (shown in Fig. 2h) that was indicative of viable virus post their release into the culture. It is noteworthy that the results of both within the cell and the culture filtrate are comparable with respect to the impact of ASS1 silencing on viral titer.

To determine whether the antiviral effect of ASS1 knockdown could be attributed specifically to reduced arginine availability, we performed rescue experiments by supplementing the culture medium with exogenous arginine (0.1 mM). Huh7 cells were treated with dsASS1 or dsGFP control, infected with CHIKV at 0.1 MOI, and cultured in the presence or absence of arginine supplementation. Viral titers were measured by plaque assay at 12 and 24 hpi. As expected, ASS1 knockdown significantly reduced viral titers in the absence of arginine supplementation compared to dsGFP controls. However, arginine supplementation partially rescued viral replication in dsASS1-treated cells, resulting in significantly higher titers compared to unsupplemented dsASS1-treated cells (****P < 0.0001 at 24 hpi) (Fig. 2l). Arginine supplementation had a minimal effect on viral titers in dsGFP-treated cells, which retain endogenous ASS1 expression and sufficient arginine biosynthetic capacity.

Collectively, these comprehensive data demonstrate that ASS1 is upregulated during CHIKV infection and plays a critical role in supporting viral replication by maintaining cellular arginine availability. ASS1 overexpression modestly enhances viral replication, while ASS1 knockdown impairs viral yield, an effect that can be rescued by exogenous arginine supplementation, confirming the specific requirement for ASS1-mediated arginine biosynthesis in CHIKV replication.

### Dose- and time-dependent arginine paradox exists during CHIKV infection in liver-derived Huh-7 cells

**Fig 3:**
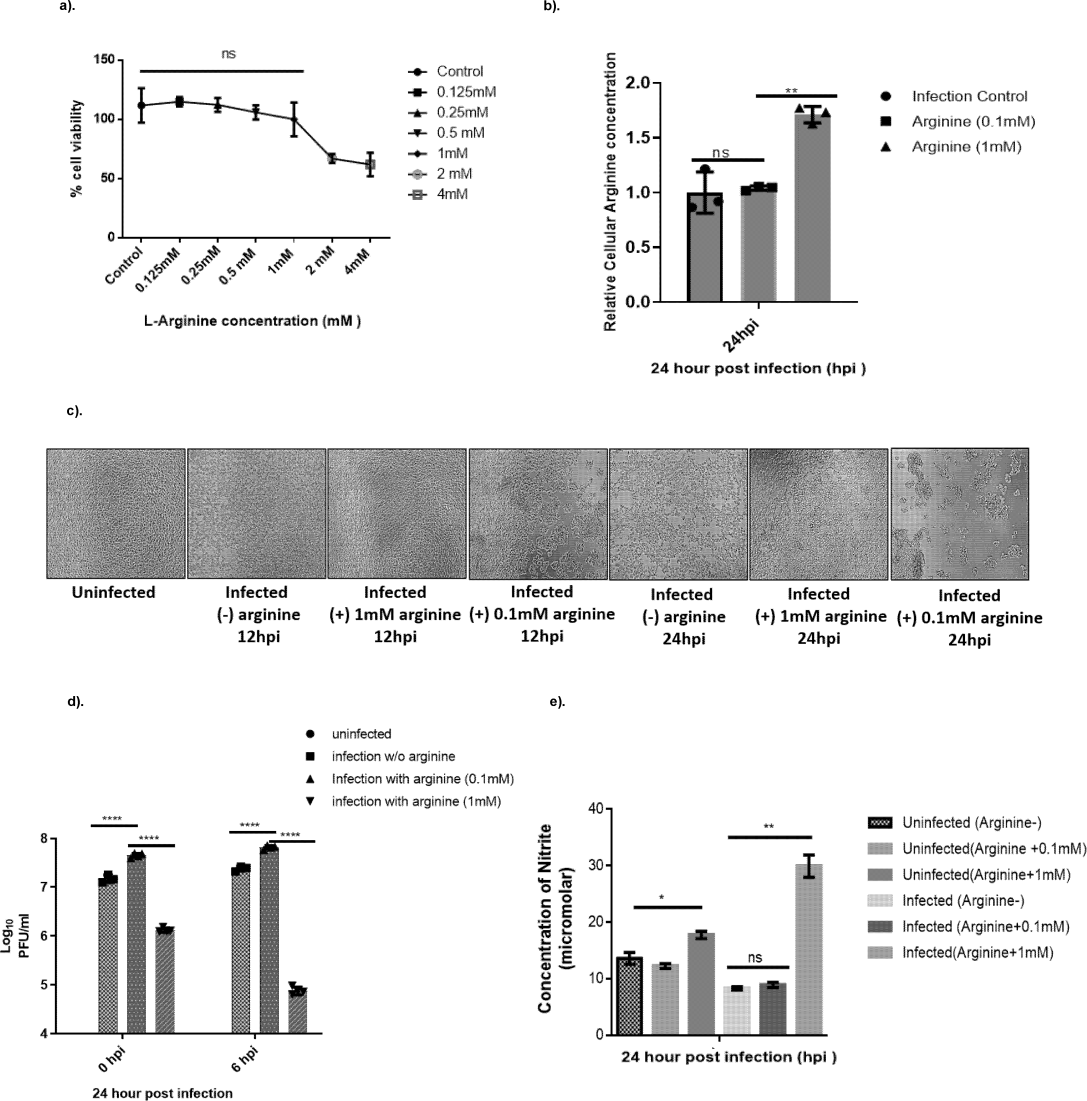
Dose-dependent arginine supplementation in CHIKV infection. Extracellular L-arginine promotes greater NO production and reduces CHIKV infection. (**a**) An MTT assay was conducted to assess the cytotoxic effects of arginine supplementation at various concentrations on Huh7 cells. The cells were cultured to 80% confluency and then treated with increasing arginine concentrations up to 4 mM. The assay followed the methods outlined in the Materials and Methods section. Error bars represent standard deviations. *P*-value significance was determined by one-way ANOVA, followed by Dunnett’s multiple comparison test, comparing each treatment with the “cells-only” control. Values with no significant difference (*P* > 0.05) by ANOVA are marked as ns. (**b**) Cellular arginine concentration was measured during exogenous arginine supplementation at 0.1 mM and 1 mM compared to the infected control. One-way ANOVA and Tukey’s multiple comparison test were used for analysis, where *P*-value: * ≤0.01, ** ≤0.001; ns represents non-significant. Error bars indicate standard deviations. (**c**) Phenotypic presentation of cytopathic effect (CPE) observed under a microscope at 10× magnification, comparing uninfected control, infected, and 0.1 mM and 1 mM arginine treated at 12 and 24 hpi. Huh7 cells were seeded in 12-well plates. Once 80% confluence was achieved, cells were infected with CHIKV at 0.1 MOI. At 0 and 6 h post-infection, 2% serum-free DMEM was replaced with L-arginine-enriched DMEM (0.1 mM and 1 mM). After 24 h, the culture filtrate was collected to measure the CHIKV titer and to visualize cytopathic effects. (**d**) The bar plot illustrates the effects of dietary L-arginine on CHIKV titers, demonstrating dose-dependent effects at 0.1 and 1 mM, as well as time-dependent effects at 0 and 6 hpi. Huh7 cells were infected with CHIKV at 0.1 MOI for 2 h and then treated with 0.1 and 1.0 mM of arginine at 0 and 6 hpi, with untreated cells serving as controls. At 24 hpi, culture filtrates were collected, and viral titers were determined by plaque assay. Data were analyzed using two-way ANOVA and Tukey’s multiple comparison test. The results are presented as means ± SD from three independent experiments, with *P* < 0.0001 ****. The group treated with 1 mM arginine is compared with the group treated with 0.1 mM arginine at 0 and 6 hpi. (**e**) Nitrite levels (μM) were measured after L-arginine supplementation (0.1 mM and 1 mM) during CHIKV infection using the Griess reagent. The experiment involved two groups, uninfected Huh7 cells and CHIKV-infected Huh7 cells, and three treatment conditions: untreated (arginine-), 0.1 mM arginine, and 1.0 mM arginine. At 24 h post-infection, nitrite levels were measured. The results are expressed as means ± SD; *P* < 0.0013**. Values not significantly different (*P* > 0.05) by ANOVA are marked as ns.

Given that CHIKV replication enhancement upon arginine supplementation was time-dependent (Fig. 1b), and previous studies have demonstrated that extracellular arginine modulates cellular processes in a dose-dependent manner, particularly affecting nitric oxide synthase (eNOS) activity (30–34), we investigated the dose-response relationship between arginine supplementation and CHIKV infection. We provided arginine at different concentrations, namely, 0.125, 0.25, 0.5, 1, 2, and 4 mM. Initial dose-ranging experiments using the above arginine concentrations revealed that concentrations ≥2 mM significantly reduced Huh-7 cell viability (Fig. 3a). Therefore, subsequent experiments focused on 0.1 mM and 1 mM arginine supplementation to avoid cytotoxic effects while examining the full range of biological responses. Intracellular arginine measurements following supplementation showed distinct concentration-dependent uptake patterns. While 0.1 mM arginine supplementation did not significantly alter intracellular arginine levels compared to infected controls, 1 mM supplementation resulted in a significant 1.7-fold increase in cellular arginine concentration (*P* = 0.0154) (Fig. 3b). These findings indicate that substantial extracellular arginine concentrations are required to significantly elevate intracellular pools in infected cells. Phenotypically, we observed concentration-dependent morphological changes. Cells supplemented with 0.1 mM arginine exhibited pronounced cytopathic effects (CPE) at 24 hpi, consistent with enhanced viral replication and associated cellular damage. In contrast, cells treated with 1 mM arginine showed minimal CPE, suggesting reduced viral activity at higher arginine concentrations (Fig. 3c). When the viral titers were evaluated upon arginine addition, we observed that 0.1 mM supplementation at 0 hpi enhanced viral replication by 3-fold compared to untreated infected controls (*P* < 0.0001). However, 1 mM arginine supplementation resulted in a dramatic 35-fold reduction in viral titers compared to 0.1 mM treatment, representing substantial inhibition of viral replication (*P* = 0.0003). Similar patterns emerged when arginine was administered at 6 hpi, although with a reduced magnitude. The 0.1 mM treatment produced a modest 2.2-fold increase in viral titers relative to untreated controls, while 1 mM supplementation caused a 99% reduction in viral titers compared to 0.1 mM treated infected cells, yielding approximately 1.0 × 10^5^ PFU/mL (Fig. 3d).

To investigate the mechanism underlying high-dose arginine inhibition of viral replication, we measured nitrite levels as a stable indicator of NO production. NO is an unstable molecule, and reliable measurements can be made of its breakdown product, nitrite (35). Simultaneously, we also evaluated nitrite levels in the above conditions and observed that endogenous NO formation is dependent on extracellular L-arginine concentration. Endogenous NO formation showed strong dependence on extracellular arginine concentration. Compared to infected controls without arginine supplementation, 1 mM arginine treatment elevated nitrite levels 4-fold during infection (*P* = 0.013), indicating substantial NO production. In contrast, 0.1 mM arginine supplementation did not significantly increase nitrite levels above control values, remaining statistically non-significant (Fig. 3e).

These results demonstrate that exogenous arginine exhibits concentration-dependent, biphasic effects on CHIKV replication: low concentrations (0.1 mM) enhance viral progeny production, while high concentrations (1 mM) inhibit viral replication through increased NO production. This phenomenon resembles the established “L-arginine paradox,” which describes how extracellular arginine influences intracellular NO levels despite intracellular arginine concentrations being sufficient to saturate eNOS under normal conditions (11). Our findings suggest that during CHIKV infection, this paradox extends to viral replication control, where the cellular response to high arginine availability shifts from supporting viral replication to activating antiviral NO-mediated responses.

### Cellular metabolome analysis reveals the importance of arginine metabolism during CHIKV infection

**Fig 4:**
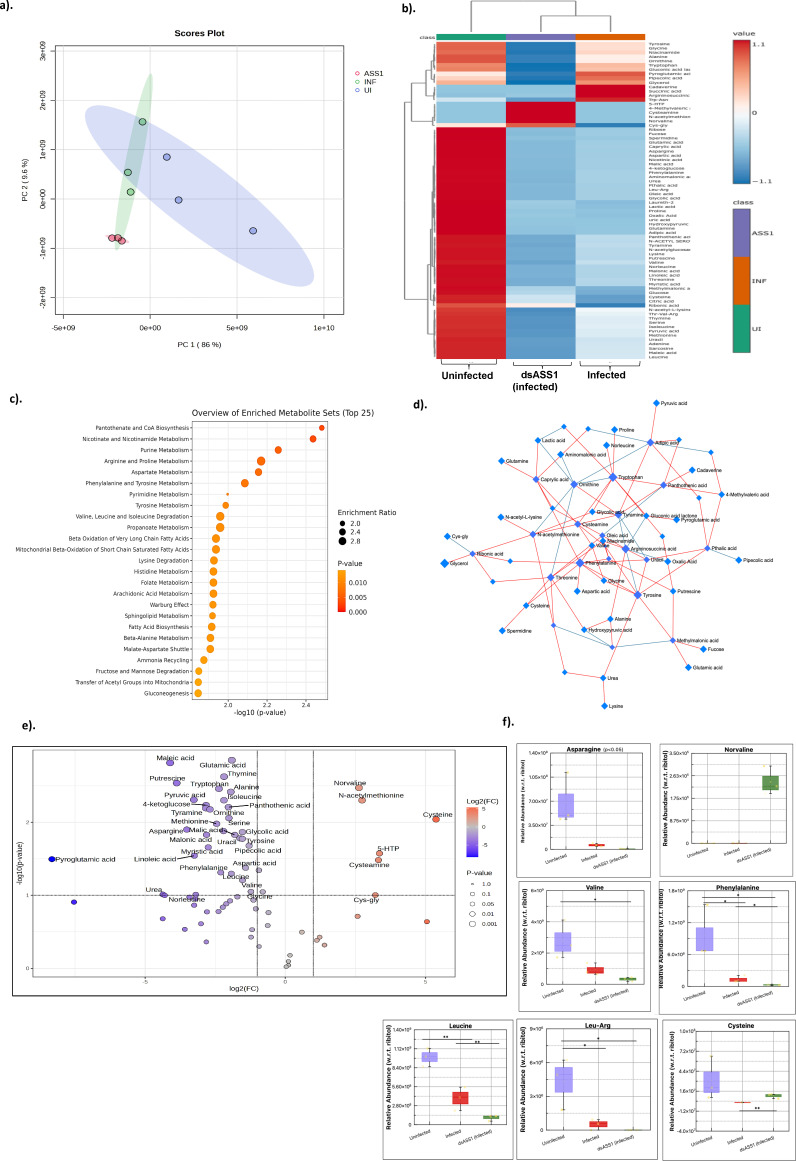
Rewiring of host cell metabolism during CHIKV infection. Metabolomic profiling and analysis of ASS1 silencing effects during infection in Huh7 cells. (**a**) Principal components analysis (PCA) was performed using normalized metabolite data from Uninfected (violet), Infected (green), and dsASS1 + Infected groups (red). The x-axis shows the first principal component, and the y-axis shows the second. Points represent experimental replicates, with colors indicating different groups. Tighter clustering within a group indicates greater separation between groups, signifying more reliable results. (**b**) Hierarchical clustering heatmap of differential metabolites, showing relative content with colors where red indicates high expression (darker shades for even higher levels) and blue indicates low expression (darker shades for lower levels). Rows display metabolite names, columns show samples, and the cluster tree on the left displays clusters of differential metabolites. Samples are grouped as green for uninfected, red for CHIKV-infected, and purple for dsASS1+CHIKV-infected samples. (**c**) Summary plot for quantitative enrichment analysis comparing significantly upregulated pathways across all conditions, presented as a bubble plot. Each bubble represents a metabolic pathway; the larger the size, the higher the enrichment ratio, and the darker the color, the greater the significance based on *P*-values. (**d**) Debiased Sparse Partial Correlation (DSPC) Network Diagram illustrating significantly altered metabolites, with each node representing a metabolite, while edges represent the correlation among them. Red lines indicate positive correlations; blue lines indicate negative correlations. (**e**) Volcano plots display metabolites with differential expression between infected and ASS1-silenced conditions, highlighting features with fold change> 2 (| log 2 FC | > 2) and *P*-value < 0. 0.1 in the *t*-test. Upregulated features are shown as red circles, downregulated as blue circles, and nonsignificant ones as gray dots. Both fold changes and *P*-values are log-transformed; points farther from (0, 0, 0) are more significant. (**f**) Box and Whisker plots depict data from different groups, with the y-axis showing the normalized relative abundance of each metabolite relative to ribitol, and the x-axis showing the groups. Metabolites identified as significant via ANOVA and Fisher’s LSD post hoc test are marked.

To further understand arginine metabolism and the impact of ASS1 during CHIKV infection, we conducted a global metabolome analysis during CHIKV infection and upon ASS1 silencing and analyzed the cellular metabolome under these conditions in comparison to the uninfected cellular state. Initial analysis of the data sets presented a tight pattern of clustering and high confidence intervals as evidenced in the PCA plot (Fig. 4a). Differential expression analysis of the identified metabolites further revealed distinct expression of metabolites that are depicted in the heatmap (Fig. 4b). Furthermore, pathway analysis of the regulated metabolites divulged regulation in pathways associated with pantothenate and CoA biosynthesis, nicotinate and nicotinamide metabolism, purine metabolism, arginine and proline metabolism, and aspartate metabolism with a high level of statistical significance (*P* < 0.01, FDR < 0.05) (Fig. 4c). The pathways that were significantly altered upon ASS1 silencing during infection include fatty acid metabolism, glutamate metabolism, Warburg effect, spermidine and spermine biosynthesis, urea cycle, glycolysis, and citric acid cycle (*P* < 0.05, FDR < 0.05). We further attempted to understand the interactions of the altered metabolites and generated a weighted node network of the metabolites in the different conditions (Uninfected, Infected, and ASS1 Silenced). The nodes that are connected by a red line indicate a positive relationship, and the nodes that are connected by a blue line indicate a negative relationship (Fig. 4d). Furthermore, we evaluated the overall differential trend in the metabolites pattern and their statistical significance using a volcano plot analysis during infection and ASS1 silencing. Our data revealed that the majority of metabolites were downregulated during ASS1 silencing (infected) compared to those that were upregulated. The most significant upregulated metabolites upon ASS1 silencing included amino acids such as cysteine, cysteamine, the arginase inhibitor norvaline, N-acetyl methionine, and the TCA cycle metabolite citric acid. However, downregulated metabolites that have a profound influence upon ASS1 silencing during CHIKV infection were amino acids such as ornithine, glutamate, putrescine, spermidine, tryptophan, asparagine, TCA cycle metabolites, that is, malic acid, and a glycolytic product such as pyruvic acid (Fig. 4e). Additionally, we also analyzed the top statistically significant amino acids abundance level upon ASS1 silencing (infection) and observed a significant reduction of asparagine (11.4-fold), leucine (3.8-fold), Leu-Arg (4.7-fold), phenylalanine (5-fold), and valine (2.8-fold) and significant increment of 6-fold in case of norvaline, cysteine (40-fold), and cysteamine (10-fold) w.r.t. infected control (Fig. 4f).

Taken together, the global metabolic changes in response to ASS1 silencing during CHIKV infection in Huh7 cells establish a potential connection between CHIKV infection and arginine metabolism at 24 hpi in Huh7 cells. The result highlights significant downregulation of important metabolites, such as argininosuccinate, as well as upregulated expression of norvaline and cysteine, suggesting involvement of pathways pertaining to glutamine, polyamines, the TCA cycle, glycolysis, and fatty acid metabolism.

### ASS1 controls CHIKV infection by modulating the generation of ROS and NO production

**Fig 5:**
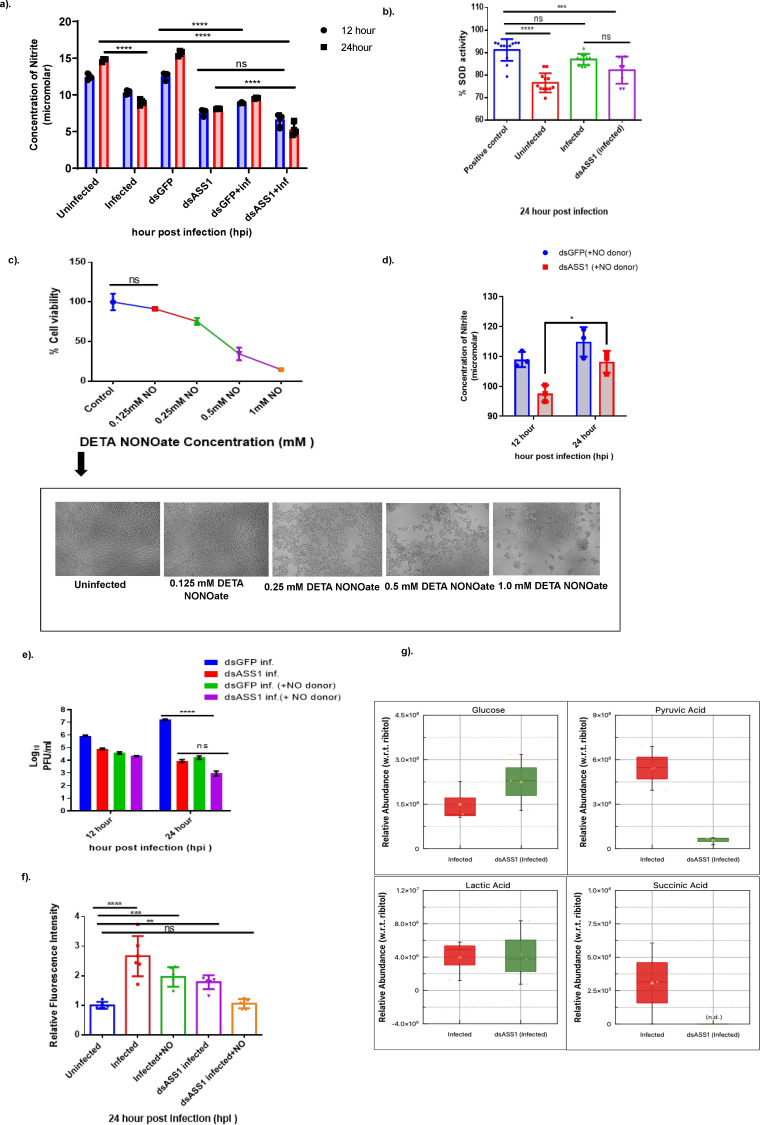
Impact of the NO donor (DETA NONOate) on CHIKV infection of Huh7 cells. (**a**) Nitrite levels in uninfected, infected, and ASS1-depleted cells were measured using Griess reagent, with absorbance at 590 nm recorded at 12 and 24 h. The experiment included uninfected Huh7 cells, Huh7 cells infected with CHIKV at 0.1 MOI, and Huh7 cells treated with dsASS1 prior to infection. At each time point and condition, nitrite levels were measured, as described in the Materials and Methods section. The experiments were repeated more than three times, and the results are presented as mean ± SD. Data were analyzed using a two-way ANOVA (*P* = 0.0001), followed by the Tukey multiple comparison test. Significant differences are marked by stars and ns for non-significant, * for *P* < 0.05, *** for *P* = 0.0001, and **** for *P* < 0.0001. (**b**) Estimation of the percentage inhibition of SOD activity in uninfected, infected, and ASS1-depleted cells at 24 h. The experiment involved uninfected Huh7 cells, Huh7 cells infected with CHIKV at 0.1 MOI, and Huh7 cells treated with dsASS1 prior to infection. At each condition, cell lysates were collected to measure SOD levels, as described in the Materials and Methods section. The graph shows the percentage inhibition based on calorimetric absorbance at 450 nm. More than five replicates were used for each condition. Data are expressed as mean ± SD and analyzed using one-way ANOVA (*P* = 0.0001) with Tukey’s multiple comparison test. (**c**) The top graph illustrates the dose-dependent impact of DETA NONOate on cell viability, measured by the MTT assay. Data are expressed as mean ± SD and analyzed using one-way ANOVA (*P* = 0.0001) with Dunnett’s multiple comparison test. The lower panel visually depicts viable cells at various DETA NONOate concentrations. (**d**) Nitrite levels were estimated 1 h after adding DETA NONOate at 12 and 24 h post-CHIKV infection in both dsGFP- and dsASS1-infected samples, using the Griess reagent. Data are shown as mean ± SD and analyzed using two-way ANOVA (*P* = 0.0001) and Tukey’s multiple-comparison test. (**e**) Quantification of virions at 12 and 24 hpi upon NO donor treatment: Huh7 cells were infected with CHIKV at 0.1 MOI, followed by the addition of DETA NONOate 1 h post-infection. At 12 and 24 hpi, the culture filtrate was collected, and infectious virions in the supernatant were measured using a plaque assay. The graph shows CHIKV titers after DETA NONOate treatment during infection control (dsGFP) and ASS1 depletion (dsASS1). Data are shown as mean ± SD and analyzed with two-way ANOVA (*P* = 0.0001), followed by Tukey’s multiple comparison test. (**f**) Bar graph showing ROS levels in uninfected Huh7 cells, infected Huh7 cells, and ASS1-depleted cells (dsASS1 + infected) at 24 h, both with and without the NO donor, DETA NONOate. The experiments were independently repeated three times. Data are presented as mean ± SD and analyzed using one-way ANOVA (*P* = 0.0001), followed by Tukey’s multiple comparison test. Significant differences are indicated by stars; ns indicates non-significance. (**g**) Metabolites identified by GC-MS are displayed as a box-and-whiskers plot comparing the infected control and dsASS1+infected conditions. The y-axis shows the relative abundance of each metabolite compared to ribitol, while the x-axis indicates the two groups. The red box represents the infected condition, and the green box indicates ASS1 silencing during infection. Two-sample *t*-tests were used to determine *P*-values. Data are shown as mean ± SD. Significant *P*-values are defined as *P* < 0.01 and *P* < 0.05. Key metabolites include glucose, pyruvate (*P* < 0.01), and lactate from glycolysis, as well as succinate (*P* < 0.05) from the TCA cycle.

One of the crucial biochemical factors that determines the intracellular availability of L-arginine for NOS is the competing enzymatic activity of enzymes of the arginine pathway (36). NO production has been reported to increase during viral infections as a host defense mechanism (37). To test the role of ASS1 in regulating ROS and NO production during CHIKV infection in Huh7 cells, we determined the levels of nitrite and superoxide dismutase (SOD) in the uninfected, infected, and ASS1-depleted cells. During infection, we recorded a reduction of 1.6-fold in nitrite level as compared to uninfected controls, whereas during ASS1 silencing, it was reduced even further to 2.7-fold (Fig. 5a). We also observed that the SOD activity during infection was quite similar to that of the positive control used to generate SOD in the kit (Fig. 5b). Although the activity was reduced in the ASS1-silenced condition, it was only marginally less than during infection. We reasoned that this could be due to the cells’ inability to produce enough NO to overcome the cellular stress caused by faulty arginine production during infection and ASS1 depletion. To address this, we exogenously provided a NO donor, namely, DETA NONOate, and studied its impact on CHIKV infection and ASS1 silencing. We estimated that DETA-NONOate was non-cytotoxic to Huh7 cells at a concentration of 0.125 mM (Fig. 5c top panel). After treating Huh7 cells with DETA-NONOate in a dose-dependent manner, we observed noticeable morphological changes and a clear reduction in cell viability as the DETA-NONOate concentration increased (Fig. 5c, lower panel). Measurement of nitrite levels in infected cells supplemented with 100 μM DETA-NONOate revealed a decrease in nitrite concentration at 12 hpi compared to the mock (GFP), followed by a gradual increase at 24 hpi (Fig. 5d). Furthermore, CHIKV titration during donor treatment showed a significant reduction in viral titer at 24 h, with 1.7 × 10^4^ PFU/mL (*P*-value 0.0001) (Fig. 5e). We reasoned that this reduction in virus titer could be attributed to the generation of ROS in the system by the NO donor. To test this hypothesis, we estimated ROS during infection and upon silencing ASS1, both with and without the addition of a NO donor. We observed a significant 3-fold increase (*P*-value <0.0001) in ROS generation during infection compared to the uninfected (control). However, following the addition of NO donor post-infection, there was a significant decline in the production of ROS. Furthermore, we also measured the ROS levels during infection combined with ASS1 silencing and found that the generation of ROS attained a level that is further incomparable to that of the uninfected control (Fig. 5f).

Interestingly, our untargeted metabolomics data also revealed a significant accumulation of succinate during CHIKV infection that could be a contributing potent driver of ROS during infection. However, upon ASS1 silencing during infection, we noted a prominent depletion in the succinate level (Fig. 5g). The analysis also showed a reduction in pyruvate levels in ASS1-silenced CHIKV-infected cells compared to infected cells. We further examined the effect of ASS1 silencing during infection on anaerobic respiratory product, that is, lactate level. Silencing of ASS1 during infection increased lactate abundance in Huh7 cells as compared to the control group, that is, CHIKV-infected Huh7 cells, indicating a switch-off of aerobic respiration to anaerobic glycolysis, thereby enhancing lactate production and highlighting the Warburg-like effect (Fig. 5g).

The above findings provide us with important clues regarding the biochemical relevance of ASS1 during CHIKV infection. First, ASS1 positively regulates NOS in Huh-7 cells, which remains maintained during CHIKV infection. Second, our results also provide evidence that ASS1 creates a nitroso-redox imbalance, thereby lowering nitrite production and promoting superoxide generation. Finally, we further demonstrate that providing a NO-rich environment through NO donors results in a significant reduction in viral titers during both infections and upon ASS1 silencing. Taken altogether, the results provide direct evidence of the role of ASS1 in regulating CHIKV infection during ROS and NO modulation in the cells.

### Arginine is diverted predominantly toward ornithine generation during CHIKV infection

**Fig 6:**
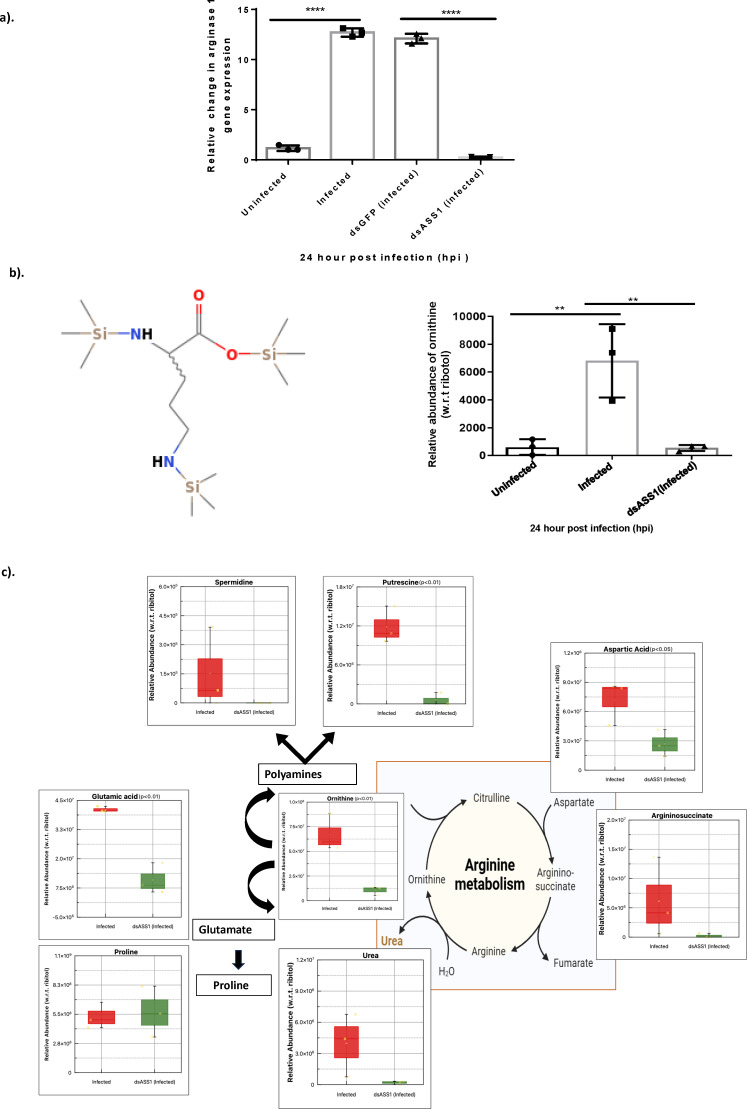
Arginase 1 expression and ornithine abundance in CHIKV-infected Huh7 cells. (**a**) Quantification of the ARG1 gene: Huh7 cells were infected with CHIKV for 24 h, then washed with PBS and lysed in TRIzol for RNA extraction. *ARG1* gene expression was amplified from the extracted RNA under different conditions and normalized to β-actin, a housekeeping gene. The relative fold change was determined using the ∆∆Ct method. One-way ANOVA and Tukey’s multiple comparison test were used for analysis and **** for *P* < 0.0001. Data are shown as mean ± SD. The experiment was performed three times. (**b**) For metabolite abundance analysis, Huh7 cells were cultured to 80% confluency and harvested at 24 hpi. After harvesting, 800 μL of chilled 80% methanol was added to stop metabolic activity. The cells were then extracted, derivatized, and the samples were prepared for GC-MS analysis. The left panel shows the chemical structure of ornithine, while the right panel illustrates the relative abundance of ornithine in infected and dsASS1 cells at 24 hpi compared to uninfected cells, as measured by GC-MS. (**c**) Quantified levels of metabolites involved in arginine metabolism and related pathways are shown. The figure illustrates metabolites biochemically linked to ASS1, L-Arg, and ARG1 during CHIKV infection and after ASS1 silencing, with detected metabolites overlaid on relevant pathways. The relative abundance is depicted as a box-and-whisker plot, with the y-axis showing metabolite levels relative to ribitol and the x-axis showing the two groups. Red boxes indicate the infected state, while green boxes indicate the dsASS1 infected state. Two-sample *t*-tests were used to determine *P*-values. Data are shown as mean ± SD. Significant metabolites are marked with *P* < 0.01 or *P* < 0.05.

Regulation of L-arginine pathways is a complex and tightly interconnected process that extends beyond the regulation of NOS or the other well-known enzyme, arginase 1 (ARG1), which competes for the common substrate, arginine. Instead, the activity of any enzyme in this pathway is influenced not only by the substrate but also by the downstream products such as ornithine, polyamines, glutamine, and proline, which collectively form an integrated metabolic network (38). To gain a better understanding, we first profiled *ARG1* expression during CHIKV infection. CHIKV-infected Huh7 cells exhibited a 7.5-fold elevation in gene expression of *ARG1* at 24 hpi relative to uninfected controls (Fig. 6, first two bars on the left), and upon silencing of ASS1, we observed a 35-fold reduction of *ARG1* expression at 24 hpi (*P*-value < 0.0001) compared to the infected (Fig. 6a, last two bars). Next, we analyzed global metabolome data to monitor metabolites biochemically linked to L-arginine, ASS1, and ARG1. We specifically looked at ornithine (Fig. 6b, left panel), as it is a key metabolite in polyamine synthesis mediated by arginase 1, and observed that ornithine abundance was significantly high (*P* value = 0.040) in infected cells as compared to uninfected cells. However, when ASS1-depleted cells were infected, ornithine levels were reduced by 12-fold with respect to infected cells (*P*-value < 0.05), attaining levels comparable to uninfected control cells (Fig. 6b right panel). We further measured the levels of metabolic products downstream of ASS1, including urea and intermediates in ornithine and polyamine metabolism, as well as amino acids such as glutamate and proline. Silencing ASS1 led to a 20-fold reduction in urea levels compared to infected samples. Since urea production relies on arginase converting arginine, decreased arginine biosynthesis probably limited substrate availability, affecting the overall metabolite level. Additionally, argininosuccinate levels declined significantly following ASS1 silencing, consistent with the impaired urea production. To investigate whether ornithine depletion upon ASS1 silencing contributes to polyamine depletion, we measured putrescine and spermidine levels and observed significant decreases of 14-fold and 11-fold, respectively (*P* < 0.01). However, it is worth noting that during CHIKV infection, spermidine levels decreased at 24 h post-infection compared with uninfected controls, probably indicating a disruption of the host’s polyamine homeostasis, potentially driven by increased viral demand for these metabolites during replication. The presence of cadaverine in infected cells and its absence upon ASS1 silencing suggest that viral infection promotes arginine-driven polyamine/diamine biosynthesis. This finding aligns with previous studies showing that CHIKV replication depends on intracellular polyamines (39). Next, to determine whether ornithine depletion might have any effect on amino acid levels such as glutamate and proline, we measured their abundance level and observed a significant 4-fold decrease in the glutamate level upon ASS1 silencing (*P*-value < 0.01), whereas changes in proline level were observed during infection and upon ASS1 silencing, although statistically not significant. Furthermore, we measured the level of the immediate upstream metabolite of ASS1, that is, aspartate, and found that there was a 2-fold decrease in its abundance level in the ASS1-silenced state (infected) as compared to the infected control (Fig. 6c).

Taken altogether, our data suggest that arginine is metabolized by ARG1 to ornithine and that there is an increase in glutamate utilization during CHIKV infection. We hypothesize that during CHIKV infection, ornithine is utilized in polyamine metabolism, and ASS1 plays a critical role in this process by regulating ARG1 expression.

### *ASS1* regulates *STAT3* activation during CHIKV infection

**Fig 7:**
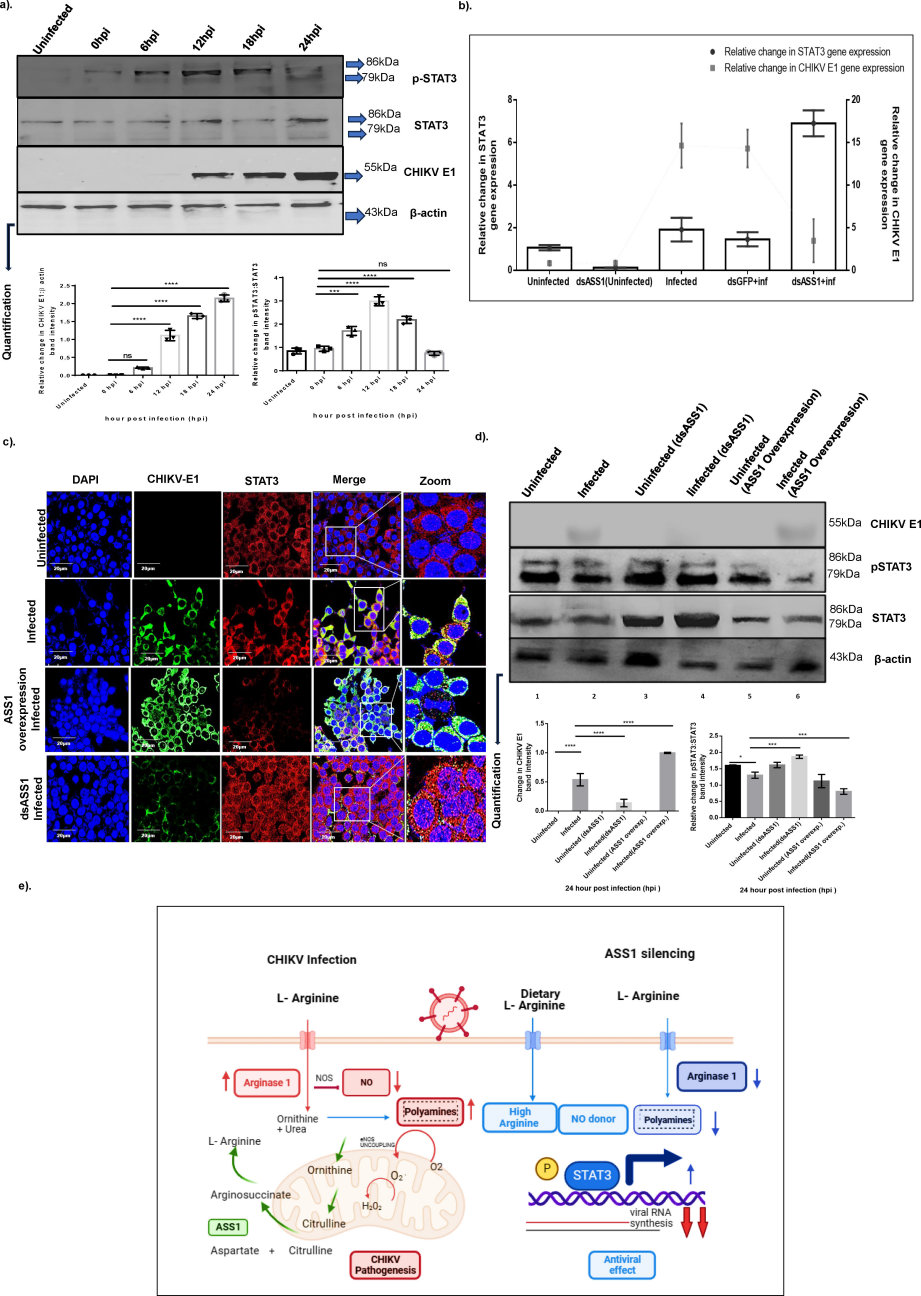
Regulation of ASS1 by *STAT3* during CHIKV infection. (**a**) Levels of STAT3, phosphorylated STAT3, and CHIKV E1 protein were quantified in CHIKV-infected Huh7 cells, with uninfected cells as controls. A representative blot from three biological replicates is shown. Protein levels were normalized to β-actin and quantified. The bar graph in the left panel shows variation in CHIKV E1 band intensity relative to β-actin across infection time points. The bar graph in the right panel displays the relative intensities of STAT3 and pSTAT3 (normalized to β-actin) during infection. Statistical significance was determined using one-way ANOVA and Tukey’s multiple comparison test, with stars indicating significant differences. (**b**) Quantification of STAT3 and CHIKV E1 genes. The genes for STAT3 and CHIKV E1 were amplified from extracted Huh7 cell RNA and normalized to β-actin, a housekeeping gene. Relative fold changes were calculated using the ∆∆Ct method. The left y-axis shows the relative change in STAT3 expression, while the right y-axis indicates the change in CHIKV genomic RNA levels. Data are presented as means ± SD from three independent experiments. Statistical significance was assessed with one-way ANOVA, with labels: ns for non-significant, * for *P* < 0.05, ** for *P* < 0.01, and *** for *P* < 0.001. (**c**) Immunofluorescence image depicting the expression of CHIKV E1 and STAT3 in Huh7 cells during uninfected, CHIKV infected, ASS1 overexpressed, and dsASS1-treated conditions. The cells were then fixed and stained with DAPI, anti-STAT3, and anti-CHIKV E1 antibodies. The merge panel displays all channels, and scale bars represent 20 µm. Representative images are shown. (**d**) Immunoblot depicting CHIKV E1, STAT3, and pSTAT3 levels at 24 h across different conditions: Uninfected, CHIKV-Infected, dsASS1 Uninfected, dsASS1 Infected, ASS1 overexpressed Uninfected, and ASS1 overexpressed Infected. β-actin served as a housekeeping control. The bar graph in the lower left panel shows the change in CHIKV E1 band intensity relative to β-actin under these conditions at 24 hpi. The right panel also displays the ratio of STAT3 to pSTAT3 band intensities across these conditions, normalized to β-actin before ratio calculation. Data analysis involved a one-way ANOVA and Tukey’s multiple comparison test; significant differences are marked by stars; ns for non-significant, * for *P* < 0.05, ** for *P* < 0.01, and *** for *P* < 0.001. Three biological replicates were performed. (**e**) A model illustrating how the arginine biosynthesis pathway is regulated during CHIKV infection.

STAT3 is reported to be activated by ASS1 during virus infection, which, in turn, promotes growth transformation (14). Furthermore, ARG1 is known to regulate polyamines and also modulate crucial signaling pathways such as mTOR and STAT3 (40). As the first indicator, we evaluated if there was any regulation of STAT3 expression during infection and if there was any change in its phosphorylation state, indicating its activation. Western blot analysis revealed basal STAT3 expression in uninfected Huh-7 cells, indicating constitutive endogenous STAT3 expression. Following CHIKV infection, phosphorylated STAT3 (pSTAT3) levels peaked during early infection time points (6 and 12 hpi) but declined sharply by 24 hpi, coinciding with increased CHIKV E1 protein expression (Fig. 7a, upper panel). Quantification across multiple experiments showed that pSTAT3 levels in infected cells decreased by approximately 52% at 24 hpi compared to 6 hpi, while total STAT3 protein levels remained largely unchanged, indicating specific suppression of STAT3 activation rather than protein degradation (Fig. 7a, lower panels). These findings align with previous reports demonstrating reduced STAT3 activation during alphavirus infections (41).

Next, to directly examine the relationship between *ASS1* and *STAT3* during CHIKV infection, we performed qRT-PCR analysis of *STAT3* gene expression under different experimental conditions at 24 hpi. ASS1 silencing alone resulted in a modest but significant decrease in basal *STAT3* expression in uninfected cells. However, during CHIKV infection, *ASS1* silencing dramatically increased *STAT3* expression by more than 3-fold compared to infected controls (*P* = 0.0026), which coincided with a significant reduction in CHIKV viral RNA levels (*P* < 0.0001) (Fig. 7b). These results suggest that *ASS1* depletion enhances antiviral *STAT3* responses that restrict viral replication.

Furthermore, to examine how ASS1 modulation affects STAT3 subcellular distribution and potential transcriptional activity, we performed immunofluorescence analysis at 24 hpi. In uninfected cells, STAT3 exhibited predominantly cytoplasmic localization with minimal nuclear presence (Fig. 7c top panel). During CHIKV infection, STAT3 expression decreased slightly and remained cytoplasmic, co-localizing with CHIKV E1 protein (Fig. 7c, panel 2). ASS1 overexpression during infection resulted in high CHIKV E1 expression, accompanied by reduced STAT3 levels, with STAT3 remaining cytoplasmic (Fig. 7c panel 3). Notably, ASS1 silencing during infection led to substantially reduced CHIKV E1 expression and promoted STAT3 nuclear translocation, where it appeared as discrete nuclear speckles characteristic of transcriptionally active STAT3 (Fig. 7c, panel 4). This nuclear accumulation pattern suggests enhanced STAT3-mediated antiviral gene transcription under ASS1-depleted conditions.

Finally, we sought to quantify how ASS1 expression levels influence STAT3 protein abundance and activation through western blot analysis, comparing different ASS1 expression states during infection at 24 h. Under basal uninfected conditions, both total STAT3 and pSTAT3 were expressed at detectable levels (Fig. 7d, immunoblot lane 1). CHIKV infection slightly reduced pSTAT3 expression while inducing prominent CHIKV E1 protein expression (Fig. 7d, immunoblot lane 2). ASS1 silencing in uninfected cells modestly increased STAT3 and pSTAT3 levels, although not significantly. However, ASS1 silencing during CHIKV infection substantially increased both total STAT3 and pSTAT3 protein levels while significantly reducing CHIKV E1 expression (Fig. 7d, immunoblot lane 4). Conversely, ASS1 overexpression during infection clearly reduced both STAT3 and pSTAT3 protein levels while enhancing CHIKV E1 expression (Fig. 7d, immunoblot lane 6). Quantification of the pSTAT3:STAT3 ratio revealed that silencing ASS1 during infection maintained higher STAT3 activation levels, 1.4-fold higher than those in control infected cells, while ASS1 overexpression suppressed STAT3 activation by 0.5-fold (Fig. 7d, 2nd graph).

Taken together, our results indicate that phosphorylation of STAT3 Y705 is observed at the initial stage of CHIKV infection, and during the course of infection, its expression declines in conjunction with an increase in CHIKV E1 expression.

Based on our comprehensive experimental evidence, we propose a working model whereby ASS1 expression modulates CHIKV infection through multiple interconnected pathways (Fig. 7e). During CHIKV infection, ASS1 upregulation contributes to enhanced viral replication by: (i) increasing arginine availability for viral protein synthesis, (ii) modulating ARG1 expression to promote ornithine and polyamine production while reducing antiviral NO synthesis through substrate competition, and (iii) suppressing STAT3 activation and nuclear translocation, thereby preventing antiviral gene expression. Conversely, ASS1 depletion restricts viral infection by (1) limiting arginine availability, (2) promoting higher arginine concentrations that favor NO production over polyamine synthesis, and (3) enhancing STAT3 activation and nuclear translocation to upregulate antiviral responses. This model illustrates how CHIKV exploits host arginine metabolism through ASS1 to create a cellular environment conducive to viral replication while simultaneously suppressing innate immune responses.

## DISCUSSION

The role of arginine metabolism in cancer and pathogenesis is well documented (7, 42–44). Among the important molecules of this pathway, ASS1 is recognized as a critical enzyme owing to its being ubiquitous yet rate-limiting in the synthesis of arginine and therefore in the downstream production of urea and NO in cells (45, 46). We have dissected some aspects of arginine metabolism during CHIKV infection in Huh7 cells and further evaluated the role of ASS1 in modulating the downstream processes of the pathway using loss-of-function and gain-of-function assays. Our study showcases for the first time how CHIKV influences ASS1 activity during infection.

Our first step toward understanding arginine metabolism during CHIKV infection was to investigate the impact of intracellular and extracellular arginine levels during infection. Note that the liver-derived Huh7 cell line that we have used has been validated as being physiologically relevant for *in vitro* CHIKV studies (47). Our observations on the effect of intracellular arginine concentrations and the timing of exogenous arginine addition on viral replication suggested that this amino acid plays a crucial role in the early events of CHIKV infection. Studies on other viruses, such as Influenza and SARS-CoV-2, have reported that derivatives of arginine could boost infection, whereas in the case of the HSV-1 virus, moderate concentrations of arginine deterred viral infection, and the measles virus was found to be conditionally dependent on arginine, with low arginine levels inhibiting the virus replication (48–50). Collectively, our results, along with other studies, emphasize the pivotal role of arginine during the early events of virus infection and its influence on the host response. It is also clear that arginine concentration, as well as the time of exposure to it, produces very different outcomes in terms of host responses and viral replication, which are dependent on the specific host-virus combination in question.

We next turned our attention to a key enzyme in cellular arginine biosynthesis, viz., argininosuccinate synthetase (ASS1). ASS1 is a rate-limiting enzyme in the citrulline-NO cycle, critical to cellular metabolism, and has been intensively studied in several disease conditions, such as cancer (4, 51, 52). Our study provides evidence of a proviral role of ASS1 during CHIKV infection in liver cells. Interestingly, exogenous supplementation of arginine at a concentration of 0.1 mM to these ASS1-depleted cells reversed the virus reduction, resulting in a significant increase in virus production. These results corroborate previous studies on the role of ASS1 in viral infection, as observed in the case of human cytomegalovirus (HCMV). However, it should be noted that the same study also found that ASS1 depletion reprogrammed the cells to be conducive to virus infection, such as HSV-1, and concluded that the activity of ASS1 toward replication was virus-specific (13).

Furthermore, we delineated the downstream processes that ASS1 dysregulation during CHIKV infection might influence, such as NO, ornithine, and polyamine generation, by exogenously supplying arginine, aspartate, and NO to host cells during infection, with or without *ASS1* silencing. We tested the impact of increased exogenous aspartate and L-arginine on the titer of CHIKV. Supplementation with arginine and aspartate enhanced virus replication, in contrast to the decrease in viral titers in *ASS1* silencing experiments. Of interest is the impact of arginine concentration on virus replication. When arginine was provided at a low concentration (0.1 mM), CHIKV infection was enhanced, as evidenced by the observed CPE, a reduction in nitrite levels, and an increase in viral titers. However, a higher arginine concentration (1 mM) did not promote virus replication. Given that the eNOS enzyme is expected to be saturated at normal intracellular concentrations of arginine, the increase in nitrate (the precursor of NO) upon exogenous arginine supplementation at 1 mM constitutes yet another instance of the “arginine paradox” (11).

By participating in the biosynthesis of arginine, ASS1 makes available an essential substrate for NO production (45). To evaluate the endogenous production of NO, we measured the nitrite level with and without ASS1 depletion. During infection, we observed a decrease in the nitrite level and an increase in SOD activity, which directly correlated with the inadequate presence of arginine. However, under ASS1-depleted conditions at 24 hpi, we observed a decrease in nitrite levels, coinciding with a reduction in CHIKV titers. Similarly, using NO donors, when we estimated the dynamics of ROS during CHIKV infection, we found no evidence of the generation of intracellular ROS that could be attributed to the reduction of viral particles in the cells. Taken together, we conclude that a synergistic effect of ROS and NO is being orchestrated by ASS1 in maintaining the nitroso-redox environment to regulate viral infection. NO production produces type 1 interferon in response to viral infections and mediates antiviral immune response. It utilizes different modes of action for virus elimination, such as nitrosylating the viral protein, inhibiting the viral enzyme, modulating transcription factors, and activating the host signaling pathway (53). The data presented in the current study suggest that during CHIKV infection, a NO donor exerts an antiviral effect, promoting virus elimination and aiding in host recovery. In some viruses, such as SARS-CoV, it has been observed that exogenous NO donors inhibit the replication cycle (54). On the other hand, certain other viruses, such as the rabies virus, utilize ROS for pathogenesis (55). NO donor concentrations appear to be crucial determinants for their beneficial or detrimental effects during *in vitro* studies.

By measuring the global metabolic changes in response to CHIKV infection and ASS1 depletion upon infection, we demonstrate the role of the crucial pathways and the role of ASS1 in modulating these pathways during CHIKV infection. Specifically, the upregulation of amino acids, such as cysteine, during ASS1 silencing may play a crucial role in mitigating oxidative stress by enhancing cellular glutathione levels, thereby acting as scavengers of reactive oxygen species (ROS) (56). Cysteine serves as a rate-limiting substrate for glutathione synthesis, and its deficiency can lead to increased lipid peroxidation and cellular damage due to ROS accumulation (43). Furthermore, studies on *Aedes* have reported that supplementation with L-cysteine in *Aedes aegypti* mosquitoes infected with CHIKV resulted in altered expression of genes involved in glutathione metabolism, suggesting a role for cysteine in managing oxidative stress during infection (57). Likewise, significant modulation in succinate levels during infection and upon ASS1 silencing highlights the critical involvement of the TCA cycle and mitochondrial electron transport chain (ETC). Accumulated succinate can disrupt ATP production, thereby reversing the electron transport chain and further enhancing ROS signaling and its generation during infection (58). Hence, maintaining a strict balance between the NO donor concentration and its impact on the generation of ROS would limit the adverse effects of NO on the host and thereby mediate antiviral immunity.

Another important enzyme involved in NO generation in the cell is ARG1. As a natural consequence of the results obtained, we studied the impact of this enzyme on nitrite levels and virus infection. A significant increase in gene expression of *ARG1* was observed at the peak of CHIKV infection, coinciding with a depletion of intracellular arginine levels. This depletion of L-arginine during infection may cause NOS uncoupling and favor the generation of superoxide over NO, as observed in our results, providing evidence of the crucial role of this enzyme in creating a metabolic environment that combats the infection (59).

Apart from acting as an endogenous regulator of nitric oxide synthase (NOS) activity via competition for their common substrate l-arginine, ARG1 is also involved in the synthesis of polyamines (60). Moreover, the genetic deletion of ARG1 also contributes to the decrease in viral loads in arthritogenic alphaviruses such as CHIKV and RRV (61). To test the role of the enzyme in this pathway during CHIKV infection, we determined the level of the precursor of polyamines, namely, ornithine, in infected and ASS1-depleted cells. We observed a direct correlation between ARG1 expression and ornithine level during infection and ASS1 silencing. A previous study has implicated polyamines in regulating Zika virus and CHIKV replication in cells by inducing the type 1 interferon pathway (62). We further proceeded to delineate the downstream processing of ornithine into polyamines and the synthesis of either proline or glutamate. Given that we did not observe any significant change in proline level and, at the same time, elevated glutamate, we reasoned that ornithine is probably being diverted toward polyamine synthesis. Earlier reports that confirm the criticality of elevated glutamate levels for polyamine synthesis further strengthen our hypothesis (63). Furthermore, neurological complications in arboviral infections have been attributed to an increase in glutamate levels resulting in activation of the GABAergic system and inhibiting the antiviral response, although the underlying mechanisms are largely unknown (64).

Taking all these results together, we postulate that during CHIKV infection in liver cells, ASS1 diverts arginine toward the synthesis of polyamines and glutamate. The implications of these findings, mechanistic and physiological, in terms of viral RNA synthesis and replication, warrant additional studies.

Some of the findings of the present study pointed us to an important regulatory molecule, namely, STAT3. STAT3 is a multifunctional protein that performs diverse and context-dependent functions, particularly in cellular responses to stress and infection (65). Additionally, the depletion of polyamines is attributed to STAT3 activation, although the mechanistic insights regarding the impact of arginine biosynthesis on STAT3 activation are poorly understood. The present study aimed to elucidate some of the mechanistic intricacies of STAT3 function during viral infection and the role of ASS1 in this process. First, our study establishes the activation of STAT3 in the early stages of CHIKV infection in liver cells, which appears to decrease during the course of infection, a feature reported in other viruses such as Influenza (66). Furthermore, the role of CHIKV non-structural protein 2, nsP2, in inhibiting the phosphorylation of STAT 1 & STAT2, and the subsequent activation to antagonize the antiviral response has been previously reported (67). Also, alphavirus inhibits the JAK-STAT signaling, thereby suppressing the interferon response (68). Furthermore, investigation of the localization pattern of unphosphorylated STAT3 during CHIKV infection reveals the translocation of STAT3 in the nucleus, similar to other reports (69). Earlier studies have further provided evidence toward the ability of unphosphorylated STAT3 in inducing antiviral response (70). It should be noted that the cytosol-to-nuclear trafficking of STAT3 is not regulated by Y705 tyrosine phosphorylation of STAT3, unlike STAT1 (71).

Furthermore, when we investigated the role of ASS1 in STAT3 functioning at the translational level, our results also indicated that depletion of ASS1 activated STAT3 phosphorylation while its overexpression inhibited the phosphorylation of STAT3 and contributed to increased CHIKV infection (Fig. 7d). Through our study, we provide evidence, albeit indirect, of the role of ASS1 in regulating ornithine production and the subsequent STAT3 expression. It is well known that ornithine serves as the primary metabolite for polyamine synthesis. Previous studies have implicated STAT3 in innate immune signaling against viral infections (72). It has also been shown by other groups that STAT3 acts as an essential antiviral mediator, as observed in the cases of Mumps and HCV viruses, whereas it is known to play a role in IFN signaling, as reported in IAV and VACV (73, 74).

Integrating all the above findings, the study provides mechanistic insights regarding the modulation of the arginine metabolism pathway by CHIKV. Unfortunately, the inhibitors against ASS1, namely, alpha-methyl D-aspartic acid, are currently banned, thereby making *in vivo* validations impossible to conduct. Despite these caveats, the overall information obtained from this study contributes to a better understanding of CHIKV pathogenesis in liver cells and may provide strategies for the development of effective antiviral therapies against CHIKV infection.

### Limitations

We acknowledge some limitations in our current research. We compared infected control cells with infected ASS1-silenced cells but did not include uninfected ASS1-silenced controls, which would help separate infection-specific effects from general consequences of ASS1 depletion, particularly for metabolomics. We also did not perform direct STAT3 loss-of-function tests because strong STAT3 depletion impairs Huh-7 cell viability and would complicate interpretation in the context of ASS1, which was the main scope of the study. Our work relied on immortalized Huh-7 hepatoma cells, which may not fully reflect responses in primary hepatocytes or other CHIKV-relevant tissues. Finally, the metabolomics workflow was tailored to arginine-related pathways and sampled at discrete time points; hence, broader or transient metabolic changes may have been under-captured. These points motivate future work, including inducible or tissue-specific STAT3 perturbation strategies, validation in primary and tissue-relevant cell models (e.g., muscle and joint cells), and expanded, higher-resolution metabolomics.

### Conclusion

Our study supports a model in which ASS1 promotes CHIKV infection by influencing arginine metabolism. ASS1 overexpression enhances viral protein accumulation and infectious yield, whereas ASS1 depletion reduces intracellular arginine, suppresses viral RNA and protein levels, and lowers progeny virus effects partially rescued by arginine supplementation. We further note an association between ARG1 expression and ornithine abundance during infection and ASS1 knockdown. Together, these findings position ASS1 as a key metabolic regulator of CHIKV replication and provide a foundation for mechanistic and translational studies targeting the ASS1–arginine-STAT3 axis.

## MATERIALS AND METHODS

### Cell culture and virus maintenance

The hepatocellular carcinoma Huh7 cell line was obtained from ATCC (Manassas, VA, USA). Huh7 cells were used for the study between passages 5 and 9. Cells were grown in Dulbecco’s modified Eagle medium (DMEM) medium supplemented with 10%FBS, 1% penicillin, and glutamine. All cell lines were maintained in a 5% CO_2_ humidified environment at 37 °C. C6/36 cells were grown in DMEM media (Cat no: AL007A) supplemented with 10% FBS and 100 units/ml penicillin G sodium and 100 µg/ml streptomycin sulfate at 5% CO_2_, a humidified environment, at 28 °C. The Vero cell line (ATCC-CCL-81) was maintained in Dulbecco’s modified Eagle medium (DMEM) (Cat no: AL007A, HiMedia, India) supplemented with 10% FBS, penicillin/streptomycin at 37°C and 5% CO_2_. The CHIKV was isolated and purified from patient sera collected during the 2010 outbreak in India and was propagated in both C6/36 and Vero cell lines (Accession no. JF950631.1). The virus propagated in C6/36 cells was collected after 96 hpi for titer determination and subsequently used to infect Huh7 cells.

### Virus propagation and infection time points

CHIKV was inoculated into C6/36 cells (MoI 0.1) and incubated at 37°C in 5% CO_2_ until the cells exhibited a cytopathic effect (CPE). Virus-containing supernatant was collected. The CHIKV virus titer was quantified using a plaque assay. For time-series infection experiments, Huh7 cells were infected with CHIKV at an MoI of 0.1 in serum-free media, with uninfected cells used as controls. After 2 h of infection, referred to as 0 hpi in our experiments, infected cells and culture filtrate from 0 hpi were collected. The media were then changed to 2% DMEM supplemented with 2% FBS, 1% penicillin/streptomycin, and 1% L-glutamine. Other time points, such as 6, 12, 18, and 24 hpi, were collected accordingly.

### Reagents and antibodies

DETA NONOate (50 mg), Cat no: 821220, was purchased from Everon Life Sciences. L-arginine (Cat no: A5006) was purchased from Sigma Aldrich. The CHIKV-nsP3 and CHIKV E1 antibodies used in the experiments were generated in the laboratory at ICGEB (75, 76). The ASS1 monoclonal antibody was purchased from Santa Cruz (sc-365475). The β-actin antibody was purchased from Santacruz (sc-47778), India. ASS1 (D404B), STAT3 (D3Z2G), and (Tyr 705) phospho-STAT3 primary antibodies were purchased from Cell Signaling Technology, India. The anti-mouse (NB120-6808) and anti-rabbit HRP-conjugated (A6154-1ML) secondary antibodies were purchased from Novus and Sigma-Aldrich, respectively. Alexa Fluor 488 Anti-mouse (ab150113) and Alexa Fluor 594 Anti-Rabbit (ab150080) were purchased from Abcam.

### Generation of plasmid constructs

The ASS1 gene was amplified from Huh7 RNA using the primers listed in Table1 and cloned it into the pCMV 3B 4T vector with a C-terminal myc-tag. To confirm the positive constructs, RT-PCR was performed using the PrimeScript One-Step RT-PCR kit (Takara, Japan) with total RNA and gene-specific primers, followed by digestion with the restriction enzymes *BamHI* and *ECORV*.

### L-arginine supplementation in cell culture

Huh7 cells were grown in six-well plates and at 80% confluency infected with CHIKV at an MoI of 0.1. L-arginine (Cat no. A5006, Sigma-Aldrich) was supplemented to the growth medium at concentrations of 0.1 and 1 mM. Arginine was added either at 0 or 6 hpi. ASS1-depleted cell cultures were supplemented with arginine at a concentration of 0.1 mM at the time of initiating CHIKV infection (0 hpi). Culture filtrate was collected after 24 hpi for the plaque assay.

### Plaque assay

To quantify the CHIKV titer in Huh7 cell culture filtrate, plaque assays were performed using a 96-well plate. The supernatant from the transfected and infected cells was collected at the specified time points from 12-well plates and used for the assay. All samples were centrifuged to separate cell debris and then used in plaque assays in a 96-well format. Vero cells were seeded at a density of 2 × 10^4^ cells/mL in 96-well plates and incubated at 37°C with 5% CO_2_ until a confluent monolayer formed. A 10-fold dilution series was prepared, and 100 μL of each dilution was added to the wells. The plates were gently shaken several times, starting from the initial adsorption period of 1 h, while being incubated at 37°C. Then, 100 μL of carboxymethyl cellulose (CMC) medium was added to the cells in each well, and the plates were incubated at 37°C and 5% CO_2_ for the development of cytopathic effects (CPE). Once the CPE was developed, the CMC overlay was removed by gentle aspiration, and the cells were fixed by adding 100 μL of 10% formaldehyde to each well and incubating the plates for 1 h. The cells were stained using 100 μL of a 0.25% crystal violet solution for 15 min. The plates were washed and gently tapped on paper towels to remove the stain, and the plaques were then counted. The virus titer was estimated as plaque-forming units per milliliter (PFU/mL) (77).

### dsRNA preparation and transfection

*In vitro* transcription using T7 and SP6 polymerases was used for ASS1dsRNA preparation using primers listed in Table 1. dsRNA purification was done using the TRIzol method. Plasmid expressing ASS1 and empty vector were transfected separately in Huh7 cells using Jet PRIME Transfection reagent as per the manufacturer’s instructions, followed by infection at 0.1 MoI of CHIKV (post 24 h of transfection); 700 ng of dsRNA was transfected into each well of 12-well plates at 70% confluency of the cells. Furthermore, the infected cells collected at 12 h and 24 h post-infection were used for RNA isolation, western blot analysis, and confocal microscopy.

**TABLE 1:** Molecular primers used in the study

Name	Sequence (5′−3′)
ASS1 Cloning Primer- F	CCGGGCGGATCCATGTCCAGCAAAGGCTCCGTGG
ASS1 Cloning Primer – R	TAATTGCTTGATATCTTTGGCAGTGACCTTGCTCTGGAGAC
ASS1 dsRNA Primer – F	TAATACGACTCACTATAGGGGGATCCTGAAATTTGCTGAGCTGGTG
ASS1 dsRNA Primer – R	TAATACGACTCACTATAGGGTCTAGACAGCCTGAGGGAATTGATGT
GFP dsRNA Primer – F	TAATACGACTCACTATAGGGATGGTGAGCAAGGGCGAGGAGCTGT
GFP dsRNA Primer – R	TAATACGACTCACTATAGGGTTACTTGTACAGCTCGTCCATGCCG

### Western blotting

Cells were lysed in RIPA buffer with protease inhibitor. Protein concentration was determined using a BCA reagent, and a 10% SDS page was run. Proteins were transferred to a nitrocellulose membrane, and blocking was performed for 1 h in 5% BSA dissolved in 1× PBS. Primary and secondary antibodies were diluted in 2.5% BSA in PBS, and washing was performed in PBS with 0.1% Tween 20. Anti-mouse primary antibodies used were ASS1, CHIKV nsP3, E1 of CHIKV, STAT3, and Tyr705 phospho-STAT3. HRP-conjugated secondary antibodies were used. Furthermore, blots were washed, and signals were detected using the Chemidoc MP imaging system (Bio-Rad) with SuperSignal West Pico PLUS Substrate (34577, Thermo Fisher Scientific). The entire washing process was performed using PBST.

### Immunofluorescence staining

Huh7 cells were seeded on coverslips and infected with CHIKV at an MoI of 0.1. At 24 h post-infection, the DMEM medium was cleared, and coverslips were washed twice with PBS. Cells were fixed using 4% formaldehyde for 20 min at room temperature. Washing was done with PBS to remove the formaldehyde. Cells were permeabilized in ice-chilled methanol for 30 min. Samples were then blocked using 1.5% BSA in 1× PBS for 60 min at room temperature. Primary staining was done on incubating overnight at 4°C using antibodies against in-house generated CHIKV nsP3 antibody (1:100), CHIKV E1 (1:100), commercially available monoclonal ASS1 antibody (1:100), and STAT3 antibody (1:100). Following three washes in PBST with 0.1% Tween 20, coverslips were stained with anti-rabbit and anti-mouse secondary antibodies (1:1,000; ABCAM) diluted in BSA-PBS. Then, the coverslips were washed three times with PBS, followed by staining with DAPI (Invitrogen) diluted in 1% BSA-PBS. Images of the samples were taken using a Nikon confocal microscope.

### ASS1 overexpression in Huh7 cells

One microgram of pCMV(empty vector) and 1 µg of pCMV containing ASS1 were transfected into 1 million Huh7 cells. Post-transfection, cells were infected with CHIKV and collected at different time points in RIPA buffer, which was then used for western blotting.

### ASS1 knockdown in Huh7 cells

dsRNA against ASS1 was synthesized by the MegaScript IVT kit according to the manufacturer’s instructions; 700 nanograms (ng) of dsRNA were used for the knockdown of ASS1, and 24 h after dsRNA transfection, infection was performed at MoI 0.1 in Huh7 cells. The supernatant was used for plaque and NO assays, and the cells were used for qRT-PCR.

### RNA extraction and qRT-PCR

Uninfected, infected, and ASS1 dsRNA-treated cells were used for experiments. At the relevant time point of the study and conditions, cells were washed once with PBS and collected in TRIzol (Takara) Reagent for RNA extraction (78). Total RNA was isolated using the TRIzol method followed by SYBR Green-based RT-qPCR experiments. For *ASS1*, *ARG1*, *STAT3*, and *CHIKV E1* gene expression analysis during infection and silencing experiments, quantitative real-time PCR (qRT-PCR) was performed using gene-specific primers listed in Table 2. The extracted RNA was subjected to quantitative real-time PCR (qPCR) to measure the levels of specific mRNAs. Actin was taken as a housekeeping control. Data were normalized by the level of housekeeping gene expression in each sample. The qPCR data were then analyzed using the 2-ΔCt method to determine the relative expression levels of these mRNAs. All the experiments were performed in three biological and three technical replicates, and the results were statistically analyzed using GraphPad Prism 6 software. All qPCR oligonucleotide primers used for the study are listed in Table 2.

**TABLE 2:** qPCR primers used in the study

Name	Sequence (5′−3′)
ASS1-F	CTTGGGGCCAAAAAGGTGTTC
ASS1-R	ATACCTGCTCTGAAGAAACT
ARG1-F	TGGACAGACTAGGAATTGGCA
ARG1-R	CCAGTCCGTCAACATCAAAACT
STAT3- F	CATATGCGGCCAGCAAAGAA
STAT3-R	ATACCTGCTCTGAAGAAACT
CHIKV E1-F	TACCCATTTATGTGGGGC
CHIKV E1-R	GCCTTTGTACACCACGATT
Actin-F	AGAGCTACGAGCTGCCTGAC
Actin-R	AGCACTGTGTTGGCGTACAG

### MTT assay

Huh7 cells were seeded in a 96-well plate at a cell density of 20,000 cells per well and incubated at 37°C overnight. Arginine, aspartate, and NO donor in serum-free DMEM media were used at different concentrations in serum-free DMEM media for 24 h at 37°C. Furthermore, cells were washed with PBS, and MTT was added at a concentration of 0.5 mg/mL in each well. For 4 h, the cells were incubated at 37°C and then washed. Finally, to dissolve the MTT crystals, DMSO was added, and the cells were incubated for an additional 30 min at 37°C. Absorbance was taken at 570 nm to calculate the percentage viability of cells.

### NO assay

The NO concentration in the infectious supernatant was measured by estimating the NO metabolite, nitrite, according to the calorimetry assay kit using the Griess reagent from Sigma Aldrich (Cat no: CCK061-200) (13).

### Arginine assay

A L-arginine assay kit from Sigma (MAK370) was used. It is an enzyme-based assay in which l-arginine is converted into a series of intermediates, which then react with a probe, producing a stable colorimetric signal at 450 nm (A450). Uninfected, CHIKV-infected, and ASS1-silenced cells were lysed in RIPA buffer, and the supernatant was further used to measure the cellular arginine concentration. First, we prepared the arginine enzyme mixture and arginine assay buffer. We further added 10 μL of the arginine enzyme mixture to each well containing standards and samples. At the same time, a 10 μL background mix was added to the sample background well. The 96-well plate was mixed thoroughly and incubated at 37°C for 60 min in the dark. Furthermore, for each well, we prepared 100 mL of Reaction Mix by combining 50 mL of Arginine Probe Mix A with 50 mL of Arginine Probe Mix B. We mixed and added 100 µL of the Reaction Mix to all wells containing standards and samples, followed by incubation for 60 min at 37 °C, protected from light. Finally, the absorbance (A450) was measured at 450 nm in a microplate reader in endpoint mode.

### SOD activity assay

For intracellular SOD (cytosolic) activity, Huh7 cells were plated at a density of 1 million cells per well in a 12-well plate and allowed to adhere for 24 h. Post-adherence, cells were infected with CHIKV at MoI 0.1, and time points were collected at 24 hpi. Cells and supernatant were collected. For the SOD assay, further cells were washed with ice-cold PBS and lysed with ice-cold lysis buffer (0.1M Tris-HCl, pH 7.4, containing 0.5% Triton X-100, 5 mM β-mercaptoethanol, and protease inhibitor). The sample was centrifuged at 14,000 × *g* for 5 min at 4 ◦C, and the supernatant was assayed using a SOD assay kit (Sigma, CS0009) (79, 80); 20 µL of the sample solution was added to each well, and 20 µL of ultrapure H2O was added to the blank wells. After that, 200 µL of WST working solution was added to each well, then 20 µL of dilution buffer was added to blank wells, and 20 µL of enzyme working solution was added to the sample wells and blank wells. The reaction mixtures were gently shaken before being incubated at 37°C for 30 min. The inhibition activity of SOD on the reaction of xanthine oxide generating superoxide with a tetrazolium salt was then determined by measuring the absorbance of the mixtures at a wavelength of 450 nm using a microplate reader. The absorbance was measured at 450 nm. The SOD activity (inhibition %) was calculated using the following equation:


SOD activity inhibition rate %=(A−B)−(C−D)/(A−B)×100

Where:


$$\begin{array}{l} A=\text{Absorbance value of No SOD control}\\ B=\text{Absorbance value of blank}\\ C=\text{Absorbance value of sample}\\ D=\text{Absorbance value of No XO or blank} \end{array}$$

### ROS assay

For dsRNA-based NO-treated conditions, intracellular ROS levels were measured using the DCFH-DA dye. Huh7 cells were plated at a density of 50,000 cells per well in a 96-well plate and incubated for 24 h. Post-adherence, the cells were treated with ASS1 dsRNA. After 18 h of treatment, the cells were infected with CHIKV at an MoI of 0.1. Two hours after infection in the treatment condition, the media were replaced with NO-enriched media. Post 12 and 24 h of incubation, the cells were washed with PBS, and 10 μL of DCFH-DA dye was added in the dark for 30 min at 37°C. Further fluorescence intensity was measured at 485 nm excitation and 535 nm emission (79).

### Metabolite extraction

Cells were grown in cell culture plates to 80% confluency and harvested at 24 hpi. Post-harvesting, chilled methanol (80%, 800 μL) was added to quench the metabolic activities. To the cell extract, ribitol (1 μL, 0.5 mg/mL) was added as a spike in the standard, followed by vortexing the mixture at 900 RPM on the remixer (Eppendorf, USA) for 30 min at 4°C. These samples were further centrifuged at 10,000 × *g* for 10 min at 4°C, and the supernatant was transferred to a new tube and vacuum-dried at 40°C using a CentiVap vacuum concentrator (Labconco, USA). These dried metabolites were stored at −20°C till mass spectrometry analysis.

### Derivatization

To the dried samples, methoxylamine hydrochloride (Sigma, 30 μL) was added, and the mixture was briefly vortexed before incubation at 60°C and 900 RPM on a thermomixer for 1 h. Then, the methoxylated samples were silylated using N-Methyl-N-(trimethylsilyl)trifluoroacetamide (MSTFA, 60 μL) with trimethylchlorosilane (TMCS, 1%) (Sigma, USA) by incubating at 60°C and 900 RPM for 1 h. The reaction mixtures were centrifuged, and the supernatant was transferred into a glass insert containing GC vials for further GC-MS analysis. Commercial standards of the target molecules were also derivatized using the above protocol to confirm the identity of metabolites.

### GC-MS analysis

Metabolite profiling was performed on Leco GC-TOF (Leco, USA). An aliquot of the sample (1 μL) was injected onto the HP-5MS (Agilent) column in splitless mode using helium as the carrier gas at a constant flow rate of 1 mL/min. The initial oven temperature was set at 50°C for 1 min, then raised to 150°C at a rate of 10°C/min and held for 3 min. Additionally, the temperature was ramped to 300°C at a rate of 7°C/min and held for 3 min. The transfer line and ion source temperatures were maintained at 260°C, 230°C, and 230°C, respectively. The energy was −70 eV in electron impact mode. The acquisition rate and mass range were 50 spectra/second and 35–600 m/z, respectively.

### Data analysis

Chroma TOF software (4.50.8.0, Leco, USA) was used for peak picking, baseline correction, deconvolution, alignment, and integration from the raw files obtained from GC-TOF runs. The identities of the metabolic features were confirmed by matching their fragmentation patterns in the NIST library and their retention times with those from their commercial standards.

GraphPad Prism 6 for Windows was used for statistical analysis. The data are expressed as mean standard deviation (s.d.). Analysis was done using a Student’s *t*-test or two-way ANOVA. One-way ANOVA was also used for multiple-group comparisons. Statistical analyses between the two groups were performed using Student’s *t*-test. *P* < 0.05 was defined as significant for all tests.

### Metabolomics data analysis

For metabolomics data analysis, heat maps, volcano plots, and box plots were generated using the freeware software MetaboAnalyst 6.0 (https://www.metaboanalyst.ca/home.xhtml). Based on the GC-MS TOF platform, we performed an unbiased, qualitative, and quantitative metabolome analysis on three groups of samples. Uninfected samples, CHIKV-infected samples, and ASS1-silenced+infected samples. The annotation of the identified metabolites was performed by matching the fragmentation patterns of each metabolite against those in the NIST library. The related pathways were identified using an MSEA with the SMPDB database. Based on the abundance of the identified metabolites, a screening analysis of the differential metabolites was conducted, and a heat map was generated. The result was plotted as the average of the three biological replicates, which showed a significant difference in metabolite abundance between the uninfected group, CHIKV-infected group, and ASS1-silencing CHIKV-infected group. Further pathway enrichment analysis of the differentially regulated metabolites was similarly performed across the groups. The top 25 significant pathways were represented as histograms based on their high statistical significance and *P*-value (*P*< 0.05). A thorough analysis of the metabolic pathways was performed by applying a Deviated Scattered Partial Correlation (DSPC) algorithm to the normalized data. The network represents the significant metabolites as a weighted node network. Metabolite concentrations (mock- vs. ASS1-silenced CHIKV-infected cells) were normalized to ribitol and uploaded to the MetaboAnalyst website under the Statistical Analysis (one-factor) module. Analysis of the data was performed by Metaboanalyst using Student’s (two-tailed) *t*-test. Graphs were plotted to compare mock-infected vs. ASS1-silenced (infected) conditions, with *P*-values set to 0.05 or lower and fold change cutoffs of 1.5-fold or higher.

### Statistical analysis

Experiments were conducted in triplicate, and the data were expressed as the mean standard deviation. Statistical analysis of experimental data was performed using GraphPad Prism (version 6). A one-way analysis of variance with Tukey’s multiple comparison test was used for comparison among multiple groups. Significant differences were denoted as follows: ns, nonsignificant; *P < 0.05; **P < 0.01; and ***P < 0.001. Significant values (*P*-value < 0.05) are represented with an asterisk(s).

## Data Availability

The metabolomic data have been uploaded to the Mendeley database and are publicly accessible at DOI: 10.17632/txsmx6kfth.1.

## References

[1] Durante W. 2022. Targeting arginine in COVID-19-induced immunopathology and vasculopathy. Metabolites 12:240. doi:10.3390/metabo12030240 35323682 PMC8953281

[2] Kiani AK, Bonetti G, Medori MC, Caruso P, Manganotti P, Fioretti F, Nodari S, Connelly ST, Bertelli M. 2022. Dietary supplements for improving nitric-oxide synthesis. J Prev Med Hyg 63:E239–E245. doi:10.15167/2421-4248/jpmh2022.63.2S3.2766 36479475 PMC9710401

[3] Morris SM. 2002. Regulation of enzymes of the urea cycle and arginine metabolism. Annu Rev Nutr 22:87–105. doi:10.1146/annurev.nutr.22.110801.140547 12055339

[4] Haines RJ, Pendleton LC, Eichler DC. 2011. Argininosuccinate synthase: at the center of arginine metabolism. Int J Biochem Mol Biol 2:8–23. 21494411 PMC3074183

[5] Phillips MM, Sheaff MT, Szlosarek PW. 2013. Targeting arginine-dependent cancers with arginine-degrading enzymes: opportunities and challenges. Cancer Res Treat 45:251–262. doi:10.4143/crt.2013.45.4.251 24453997 PMC3893322

[6] Tosato M, Calvani R, Picca A, Ciciarello F, Galluzzo V, Coelho-Júnior HJ, Di Giorgio A, Di Mario C, Gervasoni J, Gremese E, Leone PM, Nesci A, Paglionico AM, Santoliquido A, Santoro L, Santucci L, Tolusso B, Urbani A, Marini F, Marzetti E, Landi F, Gemelli against COVID-19 Post-Acute Care Team. 2022. Effects of l-arginine plus vitamin C supplementation on physical performance, endothelial function, and persistent fatigue in adults with long COVID: a single-blind randomized controlled trial. Nutrients 14:4984. doi:10.3390/nu14234984 36501014 PMC9738241

[7] Chen CL, Hsu SC, Ann DK, Yen Y, Kung HJ. 2021. Arginine signaling and cancer metabolism. Cancers (Basel) 13:3541. doi:10.3390/cancers13143541 34298755 PMC8306961

[8] Morris SM. 2016. Arginine metabolism revisited. J Nutr 146:2579S–2586S. doi:10.3945/jn.115.226621 27934648

[9] Morris SM. 2007. The journal of nutrition 6 th amino acid assessment workshop arginine metabolism: boundaries of our knowledge 1-3. J Nutr. doi:10.1093/jn/137.6.1602S 17513435

[10] Cziráki A, Lenkey Z, Sulyok E, Szokodi I, Koller A. 2020. L-arginine-nitric oxide-asymmetric dimethylarginine pathway and the coronary circulation: translation of basic science results to clinical practice. Front Pharmacol 11:569914. doi:10.3389/fphar.2020.569914 33117166 PMC7550781

[11] Shin S, Mohan S, Fung HL. 2011. Intracellular l-arginine concentration does not determine NO production in endothelial cells: implications on the “l-arginine paradox”. Biochem Biophys Res Commun 414:660–663. doi:10.1016/j.bbrc.2011.09.112 21986532 PMC3210395

[12] Henriet E, Abou Hammoud A, Dupuy J-W, Dartigues B, Ezzoukry Z, Dugot-Senant N, Leste-Lasserre T, Pallares-Lupon N, Nikolski M, Le Bail B, Blanc J-F, Balabaud C, Bioulac-Sage P, Raymond A-A, Saltel F. 2017. Argininosuccinate synthase 1 (ASS1): A marker of unclassified hepatocellular adenoma and high bleeding risk. Hepatology 66:2016–2028. doi:10.1002/hep.29336 28646562

[13] Grady SL, Purdy JG, Rabinowitz JD, Shenk T. 2013. Argininosuccinate synthetase 1 depletion produces a metabolic state conducive to herpes simplex virus 1 infection. Proc Natl Acad Sci USA 110:E5006–E5015. doi:10.1073/pnas.1321305110 24297925 PMC3870743

[14] Li T, Zhu Y, Cheng F, Lu C, Jung JU, Gao SJ. 2019. Oncogenic kaposi’s sarcoma-associated herpesvirus upregulates argininosuccinate synthase 1, a rate-limiting enzyme of the citrulline-nitric oxide cycle, to activate the STAT3 pathway and promote growth transformation. J Virol 93:30463977. doi:10.1128/JVI.01599-18 PMC6364034 30463977

[15] Ganesan VK, Duan B, Reid SP. 2017. Chikungunya virus: pathophysiology, mechanism, and modeling. Viruses 9:368. doi:10.3390/v9120368 29194359 PMC5744143

[16] Silva LA, Dermody TS. 2017. Chikungunya virus: epidemiology, replication, disease mechanisms, and prospective intervention strategies. J Clin Invest 127:737–749. doi:10.1172/JCI84417 28248203 PMC5330729

[17] Solignat M, Gay B, Higgs S, Briant L, Devaux C. 2009. Replication cycle of chikungunya: a re-emerging arbovirus. Virology (Auckl) 393:183–197. doi:10.1016/j.virol.2009.07.024 PMC2915564 19732931

[18] Bouraï M, Lucas-Hourani M, Gad HH, Drosten C, Jacob Y, Tafforeau L, Cassonnet P, Jones LM, Judith D, Couderc T, Lecuit M, André P, Kümmerer BM, Lotteau V, Desprès P, Tangy F, Vidalain P-O. 2012. Mapping of Chikungunya virus interactions with host proteins identified nsP2 as a highly connected viral component. J Virol 86:3121–3134. doi:10.1128/JVI.06390-11 22258240 PMC3302312

[19] Fros JJ, Domeradzka NE, Baggen J, Geertsema C, Flipse J, Vlak JM, Pijlman GP. 2012. Chikungunya virus nsP3 blocks stress granule assembly by recruitment of G3BP into cytoplasmic foci. J Virol 86:10873–10879. doi:10.1128/JVI.01506-12 22837213 PMC3457282

[20] Strauss JH, Strauss EG. 1994. The alphaviruses: gene expression, replication, and evolution. Microbiol Rev 58:491–562. doi:10.1128/mr.58.3.491-562.1994 7968923 PMC372977

[21] Abraham R, McPherson RL, Dasovich M, Badiee M, Leung AKL, Griffin DE. 2020. Both ADP-ribosyl-binding and hydrolase activities of the alphavirus nsP3 macrodomain affect neurovirulence in mice. mBio 11:e03253-19. doi:10.1128/mBio.03253-19 32047134 PMC7018654

[22] Schwartz O, Albert ML. 2010. Biology and pathogenesis of chikungunya virus. Nat Rev Microbiol 8:491–500. doi:10.1038/nrmicro2368 20551973

[23] Javelle E, Ribera A, Degasne I, Gaüzère BA, Marimoutou C, Simon F. 2015. Specific management of post-chikungunya rheumatic disorders: a retrospective study of 159 cases in reunion island from 2006-2012. PLoS Negl Trop Dis 9:e0003603. doi:10.1371/journal.pntd.0003603 25760632 PMC4356515

[24] Priya R, Patro IK, Parida MM. 2014. TLR3 mediated innate immune response in mice brain following infection with Chikungunya virus. Virus Res 189:194–205. doi:10.1016/j.virusres.2014.05.010 24905288

[25] Srivastava P, Kumar A, Hasan A, Mehta D, Kumar R, Sharma C, Sunil S. 2020. Disease resolution in chikungunya—what decides the outcome? Front Immunol 11:695. doi:10.3389/fimmu.2020.00695 32411133 PMC7198842

[26] Mishra N, Chaudhary Y, Chaudhary S, Singh A, Srivastava P, Sunil S. 2025. Proteomic analysis of CHIKV-nsP3 host interactions in liver cells identifies novel interacting partners. Int J Mol Sci 26:6832. doi:10.3390/ijms26146832 40725077 PMC12294820

[27] Szlosarek PW. 2014. Arginine deprivation and autophagic cell death in cancer. Proc Natl Acad Sci USA 111:14015–14016. doi:10.1073/pnas.1416560111 25228774 PMC4191809

[28] Chew HY, Cvetkovic G, Tepic S, Wells JW. 2024. Arginase-induced cell death pathways and metabolic changes in cancer cells are not altered by insulin. Sci Rep 14:4112. doi:10.1038/s41598-024-54520-z 38374190 PMC10876525

[29] Suraweera A, Münch C, Hanssum A, Bertolotti A. 2012. Failure of amino acid homeostasis causes cell death following proteasome inhibition. Mol Cell 48:242–253. doi:10.1016/j.molcel.2012.08.003 22959274 PMC3482661

[30] Mariotti F, Petzke KJ, Bonnet D, Szezepanski I, Bos C, Huneau J-F, Fouillet H. 2013. Kinetics of the utilization of dietary arginine for nitric oxide and urea synthesis: insight into the arginine-nitric oxide metabolic system in humans. Am J Clin Nutr 97:972–979. doi:10.3945/ajcn.112.048025 23535108

[31] Harrison DG. 1997. Cellular and molecular mechanisms of endothelial cell dysfunction. J Clin Invest 100:2153–2157. doi:10.1172/JCI119751 9410891 PMC508409

[32] Böger RH. 2014. The pharmacodynamics of L-arginine. Altern Ther Health Med 20:48–54. doi:10.1093/jn/137.6.1650S 24755570

[33] Ikeda K, Yamasaki H, Minami S, Suzuki Y, Tsujimoto K, Sekino Y, Irie H, Arakawa T, Koyama AH. 2012. Arginine inactivates human herpesvirus 2 and inhibits genital herpesvirus infection. Int J Mol Med 30:1307–1312. doi:10.3892/ijmm.2012.1149 23042569

[34] Rimmelzwaan GF, Baars MM, de Lijster P, Fouchier RA, Osterhaus AD. 1999. Inhibition of influenza virus replication by nitric oxide. J Virol 73:8880–8883. doi:10.1128/JVI.73.10.8880-8883.1999 10482647 PMC112914

[35] Michael Hart C, Kleinhenz DJ, Dikalov SI, Boulden BM, Dudley SC. 2005. The measurement of nitric oxide production by cultured endothelial cells. Meth Enzymol. doi:10.1016/S0076-6879(05)96042-4 16291257

[36] Caldwell RB, Toque HA, Narayanan SP, Caldwell RW. 2015. Arginase: an old enzyme with new tricks. Trends Pharmacol Sci 36:395–405. doi:10.1016/j.tips.2015.03.006 25930708 PMC4461463

[37] Abdul-Cader MS, Amarasinghe A, Abdul-Careem MF. 2016. Activation of toll-like receptor signaling pathways leading to nitric oxide-mediated antiviral responses. Arch Virol 161:2075–2086. doi:10.1007/s00705-016-2904-x 27233799 PMC7087267

[38] Canè S, Geiger R, Bronte V. 2025. The roles of arginases and arginine in immunity. Nat Rev Immunol 25:266–284. doi:10.1038/s41577-024-01098-2 39420221

[39] Mounce BC, Poirier EZ, Passoni G, Simon-Loriere E, Cesaro T, Prot M, Stapleford KA, Moratorio G, Sakuntabhai A, Levraud J-P, Vignuzzi M. 2016. Interferon-induced spermidine-spermine acetyltransferase and polyamine depletion restrict Zika and chikungunya viruses. Cell Host Microbe 20:167–177. doi:10.1016/j.chom.2016.06.011 27427208

[40] Niu F, Yu Y, Li Z, Ren Y, Li Z, Ye Q, Liu P, Ji C, Qian L, Xiong Y. 2022. Arginase: an emerging and promising therapeutic target for cancer treatment. Biomed Pharmacother 149:112840. doi:10.1016/j.biopha.2022.112840 35316752

[41] Simmons JD, White LJ, Morrison TE, Montgomery SA, Whitmore AC, Johnston RE, Heise MT. 2009. Venezuelan equine encephalitis virus disrupts STAT1 signaling by distinct mechanisms independent of host shutoff. J Virol 83:10571–10581. doi:10.1128/JVI.01041-09 19656875 PMC2753124

[42] Burrack KS, Morrison TE. 2014. The role of myeloid cell activation and arginine metabolism in the pathogenesis of virus-induced diseases. Front Immunol 5:428. doi:10.3389/fimmu.2014.00428 25250029 PMC4157561

[43] Gogoi M, Datey A, Wilson KT, Chakravortty D. 2016. Dual role of arginine metabolism in establishing pathogenesis. Curr Opin Microbiol 29:43–48. doi:10.1016/j.mib.2015.10.005 26610300 PMC4755812

[44] Xiong L, Teng JLL, Botelho MG, Lo RC, Lau SKP, Woo PCY. 2016. Arginine metabolism in bacterial pathogenesis and cancer therapy. Int J Mol Sci 17:363. doi:10.3390/ijms17030363 26978353 PMC4813224

[45] Husson A, Brasse-Lagnel C, Fairand A, Renouf S, Lavoinne A. 2003. Argininosuccinate synthetase from the urea cycle to the citrulline-NO cycle. Eur J Biochem 270:1887–1899. doi:10.1046/j.1432-1033.2003.03559.x 12709047

[46] Keshet R, Erez A. 2018. Arginine and the metabolic regulation of nitric oxide synthesis in cancer. Dis Model Mech 11:dmm033332. doi:10.1242/dmm.033332 30082427 PMC6124554

[47] Roberts GC, Zothner C, Remenyi R, Merits A, Stonehouse NJ, Harris M. 2017. Evaluation of a range of mammalian and mosquito cell lines for use in Chikungunya virus research. Sci Rep 7:14641. doi:10.1038/s41598-017-15269-w 29116243 PMC5677012

[48] Melano I, Kuo LL, Lo YC, Sung PW, Tien N, Su WC. 2021. Effects of basic amino acids and their derivatives on SARS-CoV-2 and Influenza-A virus infection. Viruses 13:1301. doi:10.3390/v13071301 34372507 PMC8310019

[49] Naito T, Irie H, Tsujimoto K, Ikeda K, Arakawa T, Koyama AH. 2009. Antiviral effect of arginine against herpes simplex virus type 1. Int J Mol Med 23:495–499. doi:10.3892/ijmm_00000156 19288025

[50] Romano N, Scarlata G. 1973. Amino acids requirements of measles virus in hela cells. Rep Group Adv Psychiatry 43:359–366. doi:10.1007/BF01556153 4788152

[51] Solomonson LP, Flam BR, Pendleton LC, Goodwin BL, Eichler DC. 2003. The caveolar nitric oxide synthase/arginine regeneration system for NO production in endothelial cells. J Exp Biol 206:2083–2087. doi:10.1242/jeb.00361 12756290

[52] Villa E, Ben-Sahra I. 2020. ASS1igning purine dependency to cancer. Nat Cancer 1:862–863. doi:10.1038/s43018-020-00117-0 35121954

[53] Garren MR, Ashcraft M, Qian Y, Douglass M, Brisbois EJ, Handa H. 2021. Nitric oxide and viral infection: recent developments in antiviral therapies and platforms. Appl Mater Today 22:100887. doi:10.1016/j.apmt.2020.100887 38620577 PMC7718584

[54] Akerström S, Mousavi-Jazi M, Klingström J, Leijon M, Lundkvist A, Mirazimi A. 2005. Nitric oxide inhibits the replication cycle of severe acute respiratory syndrome coronavirus. J Virol 79:1966–1969. doi:10.1128/JVI.79.3.1966-1969.2005 15650225 PMC544093

[55] Sander WJ, Fourie C, Sabiu S, O’Neill FH, Pohl CH, O’Neill HG. 2022. Reactive oxygen species as potential antiviral targets. Rev Med Virol 32:e2240. doi:10.1002/rmv.2240 33949029

[56] Hu Q, Dai J, Zhang Z, Yu H, Zhang J, Zhu X, Qin Y, Zhang L, Zhang P. 2023. ASS1-mediated reductive carboxylation of cytosolic glutamine confers ferroptosis resistance in cancer cells. Cancer Res 83:1646–1665. doi:10.1158/0008-5472.CAN-22-1999 36892426

[57] Kumar A, Shrinet J, Sunil S. 2023. Chikungunya virus infection in Aedes aegypti is modulated by L-cysteine, taurine, hypotaurine and glutathione metabolism. PLoS Negl Trop Dis 17:e0011280. doi:10.1371/journal.pntd.0011280 37130109 PMC10153688

[58] Murphy MP, O’Neill LAJ. 2018. Krebs cycle reimagined: the emerging roles of succinate and itaconate as signal transducers. Cell 174:780–784. doi:10.1016/j.cell.2018.07.030 30096309

[59] Roe ND, Ren J. 2012. Nitric oxide synthase uncoupling: a therapeutic target in cardiovascular diseases. Vascul Pharmacol 57:168–172. doi:10.1016/j.vph.2012.02.004 22361333

[60] Caldwell RW, Rodriguez PC, Toque HA, Narayanan SP, Caldwell RB. 2018. Arginase: a multifaceted enzyme important in health and disease. Physiol Rev 98:641–665. doi:10.1152/physrev.00037.2016 29412048 PMC5966718

[61] Stoermer KA, Burrack A, Oko L, Montgomery SA, Borst LB, Gill RG, Morrison TE. 2012. Genetic ablation of arginase 1 in macrophages and neutrophils enhances clearance of an arthritogenic alphavirus. J Immunol 189:4047–4059. doi:10.4049/jimmunol.1201240 22972923 PMC3466331

[62] Mounce BC, Cesaro T, Moratorio G, Hooikaas PJ, Yakovleva A, Werneke SW, Smith EC, Poirier EZ, Simon-Loriere E, Prot M, Tamietti C, Vitry S, Volle R, Khou C, Frenkiel M-P, Sakuntabhai A, Delpeyroux F, Pardigon N, Flamand M, Barba-Spaeth G, Lafon M, Denison MR, Albert ML, Vignuzzi M. 2016. Inhibition of polyamine biosynthesis is a broad-spectrum strategy against RNA viruses. J Virol 90:9683–9692. doi:10.1128/JVI.01347-16 27535047 PMC5068521

[63] Chattopadhyay MK, Tabor H. 2013. Polyamines are critical for the induction of the glutamate decarboxylase-dependent acid resistance system in Escherichia coli. J Biol Chem 288:33559–33570. doi:10.1074/jbc.M113.510552 24097985 PMC3837104

[64] Zhu Y, Zhang R, Zhang B, Zhao T, Wang P, Liang G, Cheng G. 2017. Blood meal acquisition enhances arbovirus replication in mosquitoes through activation of the GABAergic system. Nat Commun 8. doi:10.1038/s41467-017-01244-6 PMC5665997 29093445

[65] Johnson DE, O’Keefe RA, Grandis JR. 2018. Targeting the IL-6/JAK/STAT3 signalling axis in cancer. Nat Rev Clin Oncol 15:234–248. doi:10.1038/nrclinonc.2018.8 29405201 PMC5858971

[66] Liu S, Liu S, Yu Z, Zhou W, Zheng M, Gu R, Hong J, Yang Z, Chi X, Guo G, Li X, Chen N, Huang S, Wang S, Chen J-L. 2023. STAT3 regulates antiviral immunity by suppressing excessive interferon signaling. Cell Rep 42:112806. doi:10.1016/j.celrep.2023.112806 37440406

[67] Fros JJ, Liu WJ, Prow NA, Geertsema C, Ligtenberg M, Vanlandingham DL, Schnettler E, Vlak JM, Suhrbier A, Khromykh AA, Pijlman GP. 2010. Chikungunya virus nonstructural protein 2 inhibits type I/II interferon-stimulated JAK-STAT signaling. J Virol 84:10877–10887. doi:10.1128/JVI.00949-10 20686047 PMC2950581

[68] Simmons JD, Wollish AC, Heise MT. 2010. A determinant of Sindbis virus neurovirulence enables efficient disruption of Jak/STAT signaling. J Virol 84:11429–11439. doi:10.1128/JVI.00577-10 20739538 PMC2953173

[69] Liu L, McBride KM, Reich NC. 2005. STAT3 nuclear import is independent of tyrosine phosphorylation and mediated by importin-α3. Proc Natl Acad Sci USA 102:8150–8155. doi:10.1073/pnas.0501643102 15919823 PMC1149424

[70] Pfeffer SR, Fan M, Du Z, Yang CH, Pfeffer LM. 2017. Unphosphorylated STAT3 regulates the antiproliferative, antiviral, and gene-inducing actions of type I interferons. Biochem Biophys Res Commun 490:739–745. doi:10.1016/j.bbrc.2017.06.111 28642132 PMC5546317

[71] Reich NC, Liu L. 2006. Tracking STAT nuclear traffic. Nat Rev Immunol 6:602–612. doi:10.1038/nri1885 16868551

[72] Roca Suarez AA, Van Renne N, Baumert TF, Lupberger J. 2018. Viral manipulation of STAT3: evade, exploit, and injure. PLoS Pathog 14:e1006839. doi:10.1371/journal.ppat.1006839 29543893 PMC5854428

[73] Ulane CM, Rodriguez JJ, Parisien JP, Horvath CM. 2003. STAT3 ubiquitylation and degradation by mumps virus suppress cytokine and oncogene signaling. J Virol 77:6385–6393. doi:10.1128/jvi.77.11.6385-6393.2003 12743296 PMC155014

[74] Stevenson NJ, Bourke NM, Ryan EJ, Binder M, Fanning L, Johnston JA, Hegarty JE, Long A, O’Farrelly C. 2013. Hepatitis C virus targets the interferon-α JAK/STAT pathway by promoting proteasomal degradation in immune cells and hepatocytes. FEBS Lett 587:1571–1578. doi:10.1016/j.febslet.2013.03.041 23587486

[75] Srivastava P, Mishra N, Chaudhary S, Sunil S. 2024. Decoding chikungunya virus non-structural protein 3 interacting partners in THP-1 derived infected macrophages through proteomic profiling. Front Virol 4:1310161. doi:10.3389/fviro.2024.1310161

[76] Kumar R, Mehta D, Chaudhary S, Nayak D, Sunil S. 2022. Impact of CHIKV replication on the global proteome of Aedes albopictus cells. Proteomes 10:38. doi:10.3390/proteomes10040038 36412637 PMC9680348

[77] Sirisena PDNN, Kumar A, Sunil S. 2018. Evaluation of Aedes aegypti (diptera: culicidae) life table attributes upon chikungunya virus replication reveals impact on egg-laying pathways. J Med Entomol 55:1580–1587. doi:10.1093/jme/tjy097 29931258

[78] Rio DC, Ares M, Hannon GJ, Nilsen TW. 2010. Purification of RNA using TRIzol (TRI reagent). Cold Spring Harb Protoc 2010:pdb.prot5439. doi:10.1101/pdb.prot5439 20516177

[79] Hasan A, Devi MS S, Sharma G, Narayanan V, Sathiyarajeswaran P, Vinayak S, Sunil S. 2023. Vathasura Kudineer, an Andrographis based polyherbal formulation exhibits immunomodulation and inhibits chikungunya virus (CHIKV) under in vitro conditions. J Ethnopharmacol 302:115762. doi:10.1016/j.jep.2022.115762 36181982

[80] Yasui K, Baba A. 2006. Therapeutic potential of superoxide dismutase (SOD) for resolution of inflammation. Inflamm Res 55:359–363. doi:10.1007/s00011-006-5195-y 17122956

